# Liver Age Acceleration and Kidney Abnormalities Among Adult NHANES Participants: A Cross-Sectional Study

**DOI:** 10.3390/healthcare14213759

**Published:** 2026-11-04

**Authors:** Ruilin Wang, Baozhu Wang, Yaxin Wang, Chunxia Ren

**Affiliations:** 1 School of Nursing, Anhui Medical University, Hefei 231200, China; 2445011283@stu.ahmu.edu.cn (R.W.); 2545011419@stu.ahmu.edu.cn (B.W.); 2545011440@stu.ahmu.edu.cn (Y.W.); 2 Department of General Surgery, The First Affiliated Hospital of Anhui Medical University, Hefei 230022, China

**Keywords:** liver age acceleration, CKD, albuminuria, estimated glomerular filtration rate, biological aging, NHANES

## Abstract

**Highlights:**

**What are the main findings?**

• In a cross-sectional NHANES analysis, higher liver age acceleration (LAA5) was associated with higher odds of kidney abnormality and albuminuria after multivariable adjustment.
• Findings for reduced estimated glomerular filtration rate were less consistent across exposure specifications, adjustment models, and covariate imputation.

**What are the implications of the main findings?**

• The findings support separate assessment of albuminuria and filtration function when investigating the kidney correlates of LAA5.
• Independent prospective studies with repeated kidney assessments are needed to clarify temporal relationships and assess reproducibility.

**Abstract:**

Background: Associations of liver age acceleration (LAA5), a model-derived hepatic composite, with kidney abnormalities remain unclear. We examined associations with a composite kidney outcome and its components. Methods: This survey-weighted cross-sectional analysis included 9276 complete cases among 14,608 adults aged ≥20 years examined in the National Health and Nutrition Examination Survey (NHANES) 2017–March 2020 Pre-Pandemic and August 2021–August 2023. LAA5 represented liver age estimated using the five-marker Liver Aging Index minus chronological age. Kidney abnormality was defined as urinary albumin-to-creatinine ratio ≥ 30 mg/g or creatinine-based estimated glomerular filtration rate (eGFR) < 60 mL/min/1.73 m^2^ at one examination. Logistic regression used examination (WTMECPRP) and phlebotomy (WTPH2YR) weights, respectively, adjusting for demographic, socioeconomic, adiposity, and lifestyle factors. Results: Overall, 1521 participants had kidney abnormality. In the primary model, each standard deviation (2.85 years) higher LAA5 was associated with higher odds of kidney abnormality (odds ratio [OR], 1.16; 95% confidence interval [CI], 1.08–1.25; *p* < 0.001) and albuminuria (OR, 1.21; 95% CI, 1.13–1.30; *p* < 0.001). The continuous reduced-eGFR estimate was inconclusive (OR, 1.08; 95% CI, 0.98–1.19; *p* = 0.103), with findings varying across exposure specifications and covariate imputation. Composite and albuminuria associations persisted after additional cardiometabolic adjustment and in missing-data analyses. Spline analyses provided no clear evidence of nonlinearity. Conclusions: Higher LAA5 was associated with kidney abnormality and albuminuria. Evidence for reduced eGFR was less consistent. Prediction of future kidney disease was not assessed. Selection bias remains possible. Prospective studies with repeated kidney measurements are needed to clarify longitudinal associations.

## 1. Introduction

Chronic kidney disease (CKD) affected an estimated 788 million adults aged 20 years or older worldwide in 2023, with an age-standardized prevalence of 14.2% [1]. In the United States, the age-standardized prevalence of CKD among adults aged 20 years or older was estimated at 14.8% in 2021–2023 [2]. CKD also accounted for approximately 1.48 million deaths globally in 2023, highlighting the urgency of reducing its burden [1]. CKD and nonalcoholic fatty liver disease (NAFLD) share major cardiometabolic risk factors, and longitudinal studies have associated NAFLD with incident CKD, providing a clinical rationale for investigating liver–kidney relationships [3,4]. At the biomarker level, elastography-defined liver fibrosis has been associated with albuminuria and incident CKD [4,5], while higher gamma-glutamyl transferase (GGT) has been associated with albuminuria in nondiabetic Korean adults with preserved estimated glomerular filtration rate (eGFR) [6]. Findings for liver fat, GGT, and aminotransferases have been less consistent after cardiometabolic adjustment [4,7,8,9]. These observations support further investigation of how composite measures incorporating structural and biochemical hepatic information relate to kidney abnormalities.

The Liver Aging Index based on five liver-related markers (LAI-5) combines a fat attenuation parameter (FAP), liver stiffness measurement (LSM), alanine aminotransferase (ALT), aspartate aminotransferase (AST), and GGT [10]. In the National Health and Nutrition Examination Survey (NHANES), the fat attenuation input is the controlled attenuation parameter (CAP). CAP and LSM reflect steatosis-related attenuation and fibrosis-related stiffness, respectively [11]. Derived from sex-specific Cox–Gompertz mortality models, LAI-5 expresses modeled mortality differences in age-equivalent years; LAI-5-derived liver age acceleration (LAA5) denotes estimated liver age minus chronological age [10]. Development and validation involved adults aged 45 years or older and focused on mortality and liver-related outcomes [10]. The composite summarizes multiple hepatic measurements whose contributions differ in direction but does not directly measure biological aging or its longitudinal rate; its incremental value for kidney outcomes beyond individual components remains unestablished.

The associations of LAA5 with kidney abnormalities and their component phenotypes remain insufficiently characterized. Albuminuria and reduced eGFR represent related but distinct kidney dimensions [12,13]. Albuminuria may accompany metabolic and vascular abnormalities despite preserved filtration [12,14], whereas reduced eGFR without preceding albuminuria has been documented in type 1 diabetes [15]. An NHANES analysis found metabolic dysfunction-associated fatty liver disease (MAFLD) with fibrosis associated with albuminuria but not clearly with reduced eGFR after adjustment [16]. In Japanese men, GGT was associated with incident persistent dipstick proteinuria, whereas no clear association was detected with incident low eGFR [17]. In adults with type 2 diabetes, steatosis-related scores were associated with albuminuria progression, whereas fibrosis-related scores were associated with eGFR decline [18]. These observations support separate assessment of both renal phenotypes without presuming a stronger LAA5 association with either.

Using survey-weighted cross-sectional data from the NHANES 2017–March 2020 Pre-Pandemic and August 2021–August 2023 releases, we evaluated the primary hypothesis that higher LAA5 is associated with greater odds of kidney abnormality after multivariable adjustment in adults aged ≥20 years. The primary composite outcome was albuminuria and/or reduced eGFR detected at a single examination, without confirmation of persistence for at least 3 months [12]. Secondary analyses examined albuminuria and reduced eGFR separately; exploratory analyses assessed mutually exclusive phenotypes and potential nonlinearity. This framework evaluates cross-sectional associations without assuming a temporal or causal liver–kidney relationship.

## 2. Methods

### 2.1. Study Design and Participants

We conducted a cross-sectional analysis using data from the NHANES, including the 2017–March 2020 pre-pandemic and August 2021–August 2023 survey periods. NHANES is a nationally administered, stratified, multistage probability survey conducted by the National Center for Health Statistics (NCHS) to assess the health and nutritional status of the US civilian, noninstitutionalized population.

Demographic, questionnaire, examination, and laboratory data were linked using unique participant identifiers. Adults aged 20 years or older were eligible. To ensure the reliability of liver elastography measurements, participants were required to have an eligible FibroScan examination after fasting for at least 3 h, with at least 10 valid liver stiffness measurements and an interquartile-range-to-median ratio of 30% or less. Individuals with missing or invalid measurements required for the five-marker Liver Aging Index, serum creatinine, urinary albumin-to-creatinine ratio (UACR), or specified covariates were excluded.

**Figure 1:**
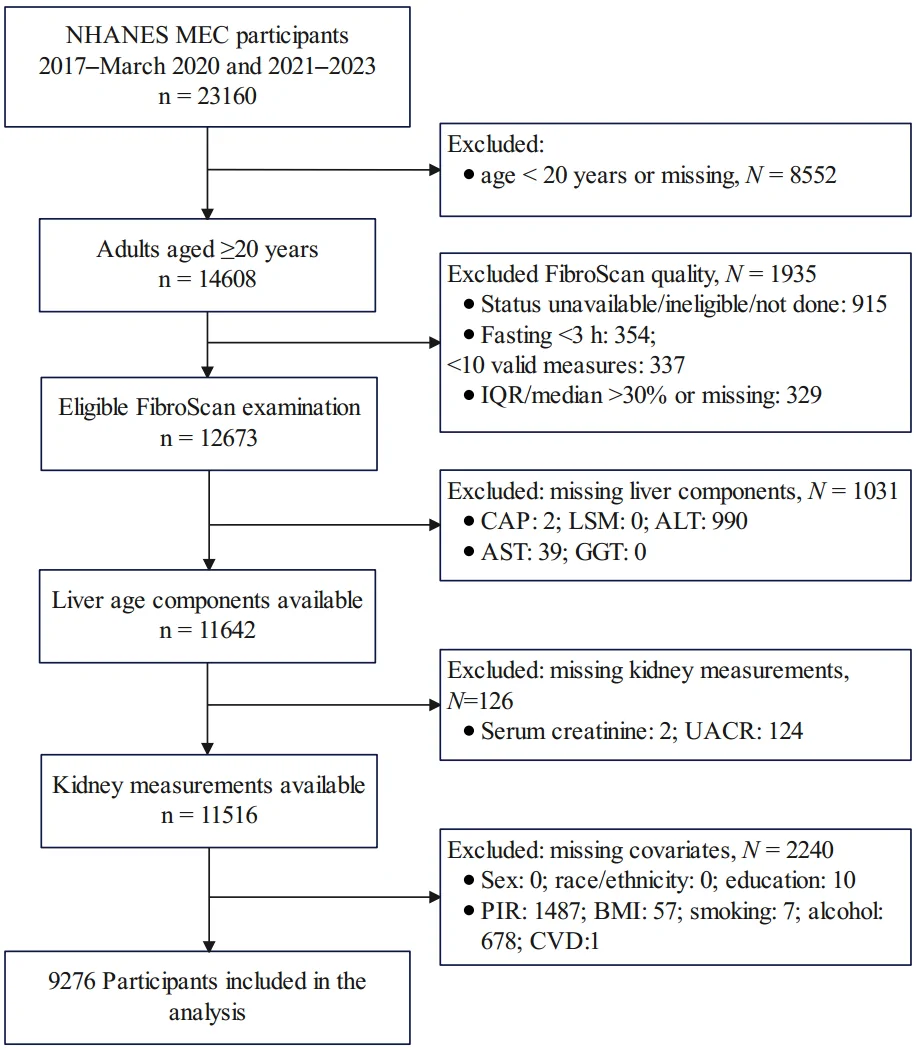
Flowchart of participant selection. Abbreviations: BMI, body mass index; CAP, controlled attenuation parameter; ALT, alanine aminotransferase; AST, aspartate aminotransferase.

The final complete-case analytical cohort comprised 9276 adults, including 5638 from 2017–March 2020 and 3638 from August 2021–August 2023. The same analytical sample was used for all three primary regression models to maintain comparability across adjustment levels. The participant selection process and reasons for exclusion are detailed in Figure 1 and Supplementary Table S1.

NHANES protocols were approved by the NCHS Research Ethics Review Board, and written informed consent was obtained from participants. This study used publicly available, deidentified data for secondary analysis and required no additional participant consent. The present secondary analysis was approved by the Clinical Research Ethics Committee of the First Affiliated Hospital of Anhui Medical University (approval no. PJ 2025-09-75; approval date: 31 October 2025).

### 2.2. Calculation of Liver Age Acceleration

LAA5 was calculated using the original LAI-5, developed from liver-related structural and biochemical measurements [10]. The five inputs were the FAP, liver stiffness measurement (LSM), alanine aminotransferase (ALT), aspartate aminotransferase (AST), and gamma-glutamyl transferase (GGT). In NHANES, FAP was operationalized using the median controlled attenuation parameter (CAP; dB/m). LSM was expressed in kPa, and ALT, AST, and GGT in U/L.

The index was implemented using the original authors’ sex-specific Cox–Gompertz calculator, with no coefficient refitting, modification, or recalibration in NHANES. Each biomarker was natural-log transformed and standardized using the original reference means and standard deviations:

(1) Z_j_ = [ln (X_j_) − μ_j_]/σ_j_

where X_j_ denotes biomarker j, and μ_j_ and σ_j_ represent the reference mean and standard deviation on the natural-log scale.

The standardized biomarkers were further centered using sex-specific constants, and their contributions were calculated using the original sex-specific coefficients [10]:

(2) S_s_ = Σ_j=1_^5^ β_j_,_s_ (Z_j_ − c_j_,_s_)

where s denotes sex, β_j_,_s_ is the sex-specific biomarker coefficient, and c_j_,_s_ is the corresponding centering constant.

The difference between the liver-model and age-only Cox–Gompertz linear predictors was then converted into an age-equivalent measure using the original sex-specific mortality rate doubling time (MRDT) [10]:

(3) LAA5_s_ = [MRDT_s_/ln (2)] × (ηliver,_s_ − ηage,_s_)

Both predictors incorporated chronological age using sex-specific centering parameters and different age coefficients; consequently, the age contribution does not cancel completely when their difference is calculated. Sex determines the applicable model coefficients, centering constants, and MRDT. The estimated LAI-5 liver age was calculated as chronological age plus LAA5.

All model coefficients, reference transformation parameters, sex-specific biomarker-centering constants, age-centering values, and MRDT parameters were applied unchanged. Complete sex-specific equations and numerical parameters are provided in the Supplementary Methods. The reference transformation parameters, sex-specific coefficients and centering constants, age coefficients, and MRDT values are summarized in Supplementary Table S2.

The primary continuous exposure was expressed per fixed 1-standard-deviation (SD) increase in LAA5, corresponding to 2.851485508 years. For categorical analyses, pooled unweighted quartile boundaries were −2.764865575, −1.116115646, and 0.515175282 years. The lowest quartile served as the reference. Quartile categories indicate relative positions within the study distribution and were not interpreted as clinically validated thresholds or absolute categories of accelerated or decelerated liver aging. Because chronological age contributes to the original liver aging index, we retained age in the regression adjustment sets to account for residual age-related variation in LAA5 and the strong age dependence of kidney outcomes. Alternative age specifications were examined in sensitivity analyses.

### 2.3. Assessment of Kidney Outcomes

eGFR was calculated from serum creatinine using the race-free 2021 Chronic Kidney Disease Epidemiology Collaboration (CKD-EPI) creatinine equation [19]. Serum creatinine was expressed in mg/dL. Public-use age values top-coded at 80 years were retained as released.

The primary outcome was laboratory-defined kidney abnormality, defined by UACR ≥ 30 mg/g or eGFR < 60 mL/min/1.73 m^2^ [12]. Secondary outcomes included albuminuria (UACR ≥ 30 mg/g) and reduced eGFR (<60 mL/min/1.73 m^2^), which were examined separately.

We also defined four mutually exclusive kidney phenotypes: neither abnormality, albuminuria only, reduced eGFR only, and both abnormalities. These phenotypes were examined descriptively and in an exploratory multinomial regression analysis.

Because UACR and serum creatinine were measured at a single examination, persistence of kidney abnormalities for at least 3 months could not be established. Accordingly, these outcomes were described as laboratory-defined kidney abnormalities rather than clinically confirmed chronic kidney disease [12].

### 2.4. Covariate Assessment

Covariates were selected according to their potential associations with liver-related biomarkers, kidney outcomes, demographic characteristics, and cardiometabolic health.

Demographic covariates included chronological age, sex, race/ethnicity, education, and poverty income ratio (PIR). PIR was categorized as <1.3, 1.3 to <3.5, or ≥3.5 for analysis. Body mass index (BMI) was calculated as weight in kilograms divided by height in meters squared.

Smoking status was categorized as never, former, or current smoking. Alcohol consumption was classified as never, former, or current drinking. The original light-, moderate-, and heavy-drinking categories were combined as current drinking for multivariable adjustment, while heavy alcohol consumption was retained as a separate restriction criterion in sensitivity analyses.

Diabetes status was classified as no diabetes, prediabetes, or diabetes according to the established algorithm incorporating available questionnaire responses, laboratory measurements, and medication information. Impaired fasting glucose or impaired glucose tolerance was classified within the prediabetes category rather than automatically treated as diabetes.

Hypertension was defined using the established questionnaire and antihypertensive medication criteria or measured systolic blood pressure ≥ 130 mmHg or diastolic blood pressure ≥ 80 mmHg [20].

Cardiovascular disease (CVD) was defined as self-reported congestive heart failure, coronary heart disease, angina, myocardial infarction, or stroke. For exploratory cardiometabolic disease-burden analyses, additional indicators distinguished confirmed absence of disease from missing or uncertain status. Uncertain status was not automatically classified as disease absence.

### 2.5. Complex Survey Design and Sampling Weights

Continuous variables were expressed as means with standard deviations or medians with interquartile ranges, and categorical variables as frequencies and percentages. Group differences were assessed using appropriate parametric or nonparametric tests.

Associations between LAA5 and kidney abnormalities were examined using survey-weighted logistic regression. Three models were constructed: Model 1 adjusted for age, sex, and race/ethnicity; Model 2 additionally adjusted for education, PIR, body mass index, smoking, and alcohol consumption; and Model 3 further adjusted for diabetes, hypertension, and CVD. Model 2 was considered the primary multivariable model. Alternative assumptions concerning the roles of diabetes, hypertension, and CVD are illustrated in Supplementary Figure S1A,B. The diagrams represent hypothesized relationships and do not establish temporal ordering or a sufficient causal adjustment set. Model 3 was interpreted as an expanded conditional-association model; neither Model 2 nor Model 3 was interpreted as identifying a total or direct causal effect [21].

Associations were reported as odds ratios (ORs) with 95% confidence intervals (CIs) per 1-SD increase in LAA5 and across quartiles, with the lowest quartile as the reference. Trends across quartiles were evaluated using ordinal scores. Restricted cubic splines with three knots at the 10th, 50th, and 90th percentiles were used to examine nonlinear associations, with the median LAA5 as the reference [22]. The three-knot specification was used to allow departures from linearity while limiting model complexity. Overall and nonlinear associations were evaluated using design-adjusted joint Wald tests. Four-knot models were examined as a sensitivity analysis.

Subgroup analyses were conducted according to age, sex, body mass index, diabetes, hypertension, survey period, and CVD, with interactions assessed using multiplicative terms. Subgroup differences were evaluated using interaction tests rather than comparisons of within-subgroup statistical significance [23]. Cardiometabolic disease burden was additionally categorized as 0, 1, or 2–3 conditions, with participants whose disease burden could not be determined excluded from these analyses. Exploratory multinomial logistic regression was performed for four mutually exclusive kidney phenotypes: neither abnormality, albuminuria only, reduced eGFR only, and both abnormalities. Relative odds ratios (RORs), overall heterogeneity tests, and pairwise contrasts were estimated. Benjamini–Hochberg correction was applied to subgroup and disease-burden interaction tests, and Holm correction was applied to multinomial pairwise comparisons.

Sensitivity analyses included additional adjustment for lipid parameters or lipid-lowering medications, substitution of waist circumference for body mass index, flexible age adjustment, exclusion of heavy alcohol consumers, restriction to participants aged ≥45 years or with confirmed negative viral hepatitis status, alternative sampling weights, and recalculation of eGFR using the 2009 CKD-EPI equation [24]. Prevalence ratios (PRs) were additionally estimated using modified Poisson regression. Associations of the five individual liver biomarkers with kidney abnormalities were also examined.

The primary analysis was based on complete cases. Multiple imputation was performed among 11,516 eligible participants with observed exposure and outcomes, generating 20 imputed datasets for missing covariates. Response-probability-weighted analyses were additionally conducted to assess potential selection bias [25]. All analyses were performed using R version 4.6.1. Statistical tests were two-sided, with *p* < 0.05 considered statistically significant. Exploratory findings were interpreted considering multiple testing.

## 3. Results

### 3.1. Participant Characteristics

A total of 23,160 NHANES mobile examination center (MEC) participants were initially identified across the two survey periods. Among 14,608 adults aged 20 years or older, 11,516 had the required liver and kidney measurements. After excluding participants with incomplete covariate information, 9276 adults were included in the final analysis, comprising 5638 participants from 2017–March 2020 and 3638 from August 2021–August 2023 (Figure 1 and Supplementary Table S1). The mean age was 48.19 (SD, 16.91) years, and 4845 participants were women (weighted proportion, 50.6%). The survey-weighted mean LAA5 was −1.22 (SD, 2.76) years, with a weighted median of −1.27 years (interquartile range, −2.92 to 0.34) and an observed range of −12.27 to 21.28 years.

Baseline characteristics differed across LAA5 quartiles. Significant differences were also observed in sex, race/ethnicity, PIR, BMI, smoking status, diabetes, hypertension, and CVD (all *p* < 0.05), whereas educational attainment and alcohol consumption did not differ significantly across quartiles. Mean age was 51.31 years in Q1 and 45.35 years in Q4, while median CAP was 297.0 and 219.0 dB/m, respectively. Liver-related measurements also varied across the LAA5 distribution, with higher median liver stiffness and GGT levels in Q4 than in Q1 (Table 1).

Overall, 1521 participants had laboratory-defined kidney abnormalities, including 1081 with albuminuria and 668 with reduced eGFR. When classified into mutually exclusive phenotypes, 7755 participants had neither abnormality, 853 had albuminuria only, 440 had reduced eGFR only, and 228 had both abnormalities; the corresponding survey-weighted proportions were 86.9%, 7.8%, 3.7%, and 1.6%, respectively (Supplementary Table S3). Survey-weighted mean LAA5 values in these four groups were −1.25, −0.80, −1.49, and −0.81 years, respectively.

The LAA5 distribution showed modest inverse age dependence, with a survey-weighted correlation of −0.113 (95% CI, −0.136 to −0.090; Supplementary Figure S2A). The overall survey-weighted density showed a longer positive tail (Supplementary Figure S2B). Weighted density estimates were broadly similar between the two survey periods (Supplementary Figure S2C), although distributions varied across mutually exclusive kidney phenotypes (Supplementary Figure S2D).

### 3.2. Association Between LAA5 and Kidney Abnormalities

Higher LAA5 was associated with greater odds of kidney abnormality across the progressively adjusted regression models (Table 2). In the primary multivariable model (Model 2), each fixed 1-SD increase in LAA5 (2.8515 years) was associated with 16% higher odds of kidney abnormality (OR, 1.16; 95% CI, 1.08–1.25; *p* < 0.001). The association persisted after additional adjustment for diabetes, hypertension, and CVD in Model 3 (OR, 1.12; 95% CI, 1.04–1.21; *p* = 0.003).

In the quartile analysis, participants in Q4 had higher odds of kidney abnormality than those in Q1 in both Model 2 (OR, 1.47; 95% CI, 1.13–1.91; *p* = 0.005) and Model 3 (OR, 1.32; 95% CI, 1.01–1.73; *p* = 0.040). The corresponding tests for trend were significant (*p* for trend = 0.003 and 0.026, respectively).

For the secondary outcomes, each 1-SD increase in LAA5 was associated with higher odds of albuminuria in Model 2 (OR, 1.21; 95% CI, 1.13–1.30; *p* < 0.001) and Model 3 (OR, 1.17; 95% CI, 1.09–1.26; *p* < 0.001). In contrast, the corresponding associations with reduced eGFR were not statistically significant in either Model 2 (OR, 1.08; 95% CI, 0.98–1.19; *p* = 0.103) or Model 3 (OR, 1.05; 95% CI, 0.96–1.15; *p* = 0.294).

For albuminuria, the Q4 versus Q1 OR was 1.52 (95% CI, 1.13–2.03; *p* = 0.007) in Model 2 and 1.34 (95% CI, 0.99–1.83; *p* = 0.059) in Model 3; the corresponding *p* values for trend were 0.003 and 0.033. For reduced eGFR, Q4 was associated with higher odds than Q1 in Model 2 (OR, 1.37; 95% CI, 1.03–1.81; *p* = 0.032; *p* for trend = 0.026), whereas the corresponding association was attenuated in Model 3 (OR, 1.26; 95% CI, 0.97–1.64; *p* = 0.085; *p* for trend = 0.072).

### 3.3. Nonlinear Associations and Individual Liver Components

**Figure 2:**
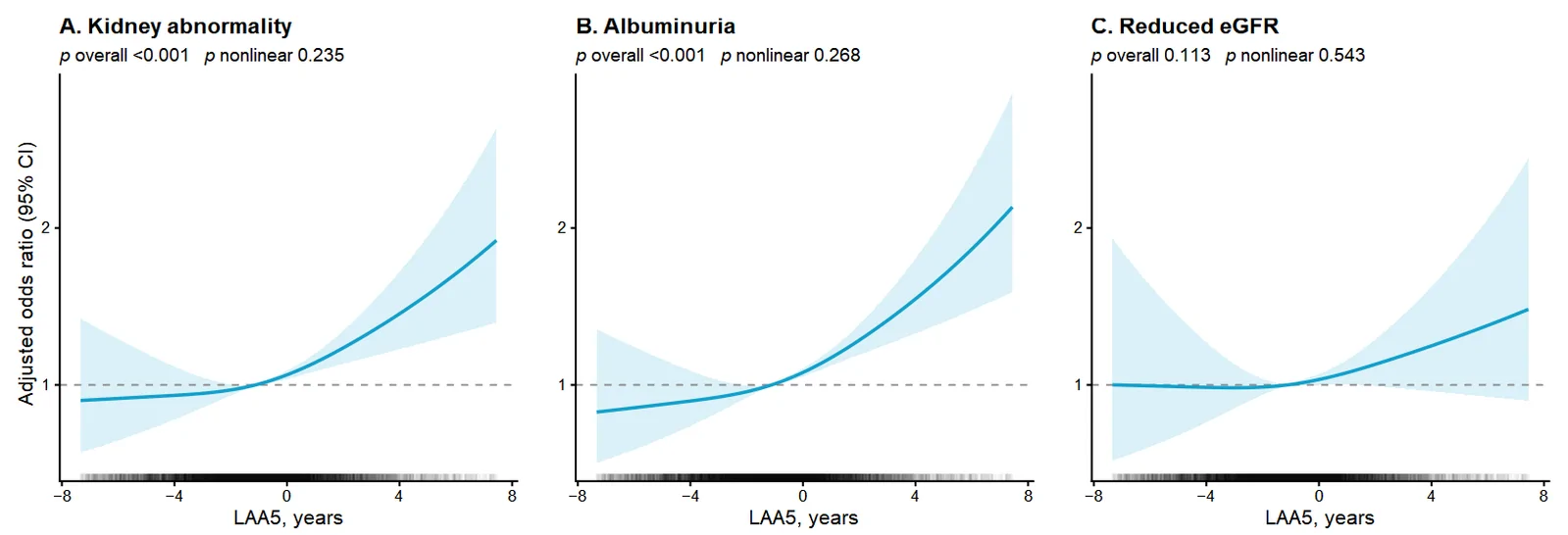
Dose–response associations of LAA5 with kidney outcomes. Restricted cubic spline analyses were used to evaluate the associations of LAI-5-derived liver age acceleration (LAA5) with kidney outcomes. Panel (**A**) shows the association between LAA5 and kidney abnormality. Panel (**B**) shows the association between LAA5 and albuminuria. Panel (**C**) shows the association between LAA5 and reduced estimated glomerular filtration rate (eGFR). Three-knot restricted cubic splines use fixed pooled P10, P50, and P90 knots and the pooled median (−1.1161 years) as reference. Curves show Model 2 adjusted ORs with 95% CIs over P1–P99; rugs show observed exposure values. Overall and nonlinear *p* values are design-adjusted joint Wald tests.

Restricted cubic spline analyses supported an overall association between LAA5 and kidney abnormality in Model 2 (*p* for overall association < 0.001), without evidence of a statistically significant departure from linearity (*p* for nonlinearity = 0.235; Figure 2A). A similar pattern was observed for albuminuria (*p* for overall association < 0.001; *p* for nonlinearity = 0.268; Figure 2B). For reduced eGFR, neither the overall association (*p* = 0.113) nor the nonlinear component (*p* = 0.543) was statistically significant (Figure 2C).

In the four-knot sensitivity analysis, overall associations remained statistically significant for kidney abnormality and albuminuria (both *p* < 0.001), without evidence of nonlinearity (*p* = 0.277 and 0.521, respectively; Supplementary Figure S3A,B). For reduced eGFR, neither the overall association (*p* = 0.109) nor the nonlinear component (*p* = 0.206) was statistically significant in the four-knot model (Supplementary Figure S3C).

**Figure 3:**
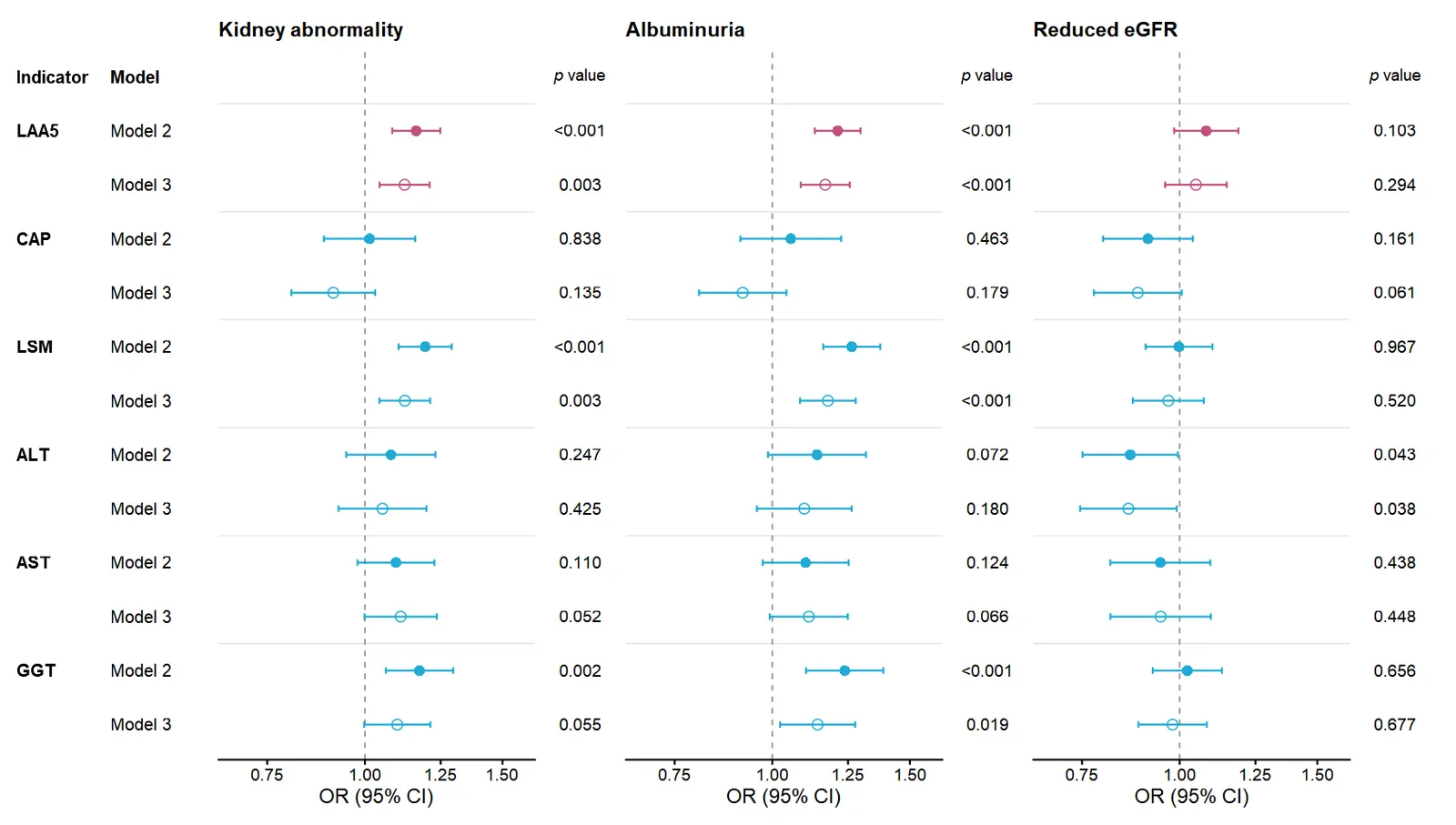
LAA5 and Individual Component Associations. Note. Each exposure is modeled separately. Filled and open points denote Models 2 and 3, respectively, also identified in the model column. Components are natural-log transformed and standardized by their own fixed SD; these are association comparisons, not tests of incremental prediction. Model 1 adjusts for age, sex, and race/ethnicity. Model 2 additionally adjusts for education, PIR (<1.3, 1.3 to <3.5, or ≥3.5), BMI, smoking (never, former, or current), and alcohol use (never, former, or current). Model 3 additionally adjusts for diabetes, hypertension, and CVD. *p* values are two-sided design-based tests for each exposure-specific slope.

In separate models of the natural-log-transformed and standardized liver components, LSM and GGT showed positive associations with kidney abnormality in Model 2 (LSM: OR, 1.19; 95% CI, 1.10–1.29; GGT: OR, 1.17; 95% CI, 1.06–1.30). After expanded adjustment in Model 3, the association remained statistically significant for LSM (OR, 1.12; 95% CI, 1.04–1.21), whereas the GGT estimate was attenuated (OR, 1.10; 95% CI, 1.00–1.21; *p* = 0.055). CAP, ALT, and AST did not show statistically significant associations with the composite kidney outcome in Model 3. For albuminuria, both LSM (OR, 1.18; 95% CI, 1.09–1.28) and GGT (OR, 1.14; 95% CI, 1.02–1.28) remained positively associated after expanded adjustment. For reduced eGFR, ALT showed an inverse association in both Model 2 and Model 3 (OR, 0.86; 95% CI, 0.75–0.99 in both models; nominal *p* = 0.043 and 0.038, respectively), whereas the other four individual components did not show statistically significant associations. These component-specific findings represent separate exposure associations rather than formal comparisons of predictive performance (Figure 3 and Supplementary Table S4).

### 3.4. Subgroup and Cardiometabolic Burden Analyses

The association between LAA5 and kidney abnormality was generally consistent across subgroups defined by age, BMI, diabetes, hypertension, survey period, and CVD, with no statistically significant interactions for these factors (all interaction *p* > 0.05; Supplementary Table S5).

A nominal sex interaction was observed (*p* for interaction = 0.027), with a stronger estimated association among women (OR, 1.33; 95% CI, 1.17–1.52) than among men (OR, 1.09; 95% CI, 0.99–1.21). However, this interaction did not remain statistically significant after false discovery rate (FDR) correction (adjusted *p* = 0.192). None of the seven subgroup interaction tests remained significant following multiple-testing adjustment.

Among 8959 participants with fully ascertained diabetes, hypertension, and CVD status, hypertension alone was the most frequent pattern involving at least one condition (*n* = 3306), and 353 participants had all three conditions (Supplementary Figure S4A). Among the patterns with displayed prevalence estimates, the survey-weighted kidney-abnormality prevalence point estimate was highest in participants with all three conditions and lowest in those with none (Supplementary Figure S4B). The estimate for diabetes plus CVD without hypertension was not displayed because of low survey design degrees of freedom (df = 5).

In exploratory analyses stratified by cardiometabolic disease burden, 8977 participants could be classified into groups with 0, 1, or 2–3 conditions, while 299 had indeterminate burden. The corresponding numbers were 3410, 3673, and 1894 participants, representing 45.0%, 38.8%, and 16.2% of the survey-weighted classifiable population, respectively. This broader classification included 18 participants with at least two confirmed conditions despite uncertainty in the third condition. The LAA5–kidney abnormality association did not differ significantly across burden categories (*p* for interaction = 0.888). Similarly, no significant burden interactions were identified for albuminuria (*p* = 0.584) or reduced eGFR (*p* = 0.842; Supplementary Table S6).

### 3.5. Exploratory Analysis of Mutually Exclusive Kidney Phenotypes

Exploratory multinomial logistic regression included 9276 participants, with neither abnormality as the reference category (Supplementary Table S7).

In Model 2, each 1-SD increase in LAA5 was associated with higher relative odds of albuminuria only (ROR, 1.20; 95% CI, 1.10–1.31) and both abnormalities (ROR, 1.28; 95% CI, 1.13–1.46), whereas the association with reduced eGFR only was not statistically significant (ROR, 1.03; 95% CI, 0.89–1.19). These patterns persisted after expanded adjustment in Model 3, with RORs of 1.16 (95% CI, 1.05–1.27), 1.22 (95% CI, 1.10–1.37), and 1.00 (95% CI, 0.87–1.16), respectively.

The direct comparison between albuminuria-only and reduced-eGFR-only coefficients yielded a ratio of RORs of 1.17 (95% CI, 1.00–1.35; nominal *p* = 0.043) in Model 2 and 1.15 (95% CI, 0.99–1.34; nominal *p* = 0.070) in Model 3. However, the overall tests of coefficient heterogeneity were not statistically significant in either model (*p* = 0.066 and 0.102, respectively).

### 3.6. Sensitivity and Missing Data Analyses

The association between LAA5 and kidney abnormality remained broadly consistent across sensitivity analyses (Supplementary Table S8 and Supplementary Figure S5). In Model 3, additional adjustment for total cholesterol and high-density lipoprotein (HDL) cholesterol (OR, 1.13; 95% CI, 1.05–1.22), adjustment for lipid-lowering medication use (OR, 1.12; 95% CI, 1.04–1.20), and replacement of BMI with waist circumference (OR, 1.12; 95% CI, 1.03–1.20) yielded estimates similar to the corresponding Model 3 estimate in the main analysis. The respective sample sizes were 9273, 9260, and 9152. Comparable results were obtained after excluding heavy alcohol consumers (*n* = 7435; OR, 1.10; 95% CI, 1.00–1.20; *p* = 0.049), restricting the analysis to participants aged 45 years or older (*n* = 5864; OR, 1.15; 95% CI, 1.05–1.26), or including only participants explicitly negative for viral hepatitis (*n* = 8976; OR, 1.10; 95% CI, 1.01–1.19). Excluding participants with confirmed viral hepatitis while retaining those with unknown status also yielded a positive association (*n* = 9034; Model 3 OR, 1.10; 95% CI, 1.01–1.19; *p* = 0.022). Flexible age adjustment also produced an unchanged Model 3 estimate (OR, 1.12; 95% CI, 1.04–1.21).

Replacing the 2021–2023 phlebotomy weight with the standard MEC weight yielded a Model 3 OR of 1.13 (95% CI, 1.05–1.21), compared with the corresponding main-analysis Model 3 OR of 1.12 (95% CI, 1.04–1.21). Recalculating eGFR using the 2009 CKD-EPI equation likewise produced a positive association (OR, 1.11; 95% CI, 1.03–1.20). The composite kidney outcome was identified in 1594 participants using the 2009 equation, compared with 1521 using the 2021 equation.

Using PRs rather than odds ratios, the composite kidney outcome remained positively associated with LAA5 in Model 2 (PR, 1.12; 95% CI, 1.06–1.18) and Model 3 (PR, 1.09; 95% CI, 1.03–1.14; Supplementary Table S9). Albuminuria was also positively associated with LAA5 in Model 2 (PR, 1.18; 95% CI, 1.11–1.24) and Model 3 (PR, 1.13; 95% CI, 1.07–1.20). The corresponding associations with reduced eGFR were not statistically significant in Model 2 (PR, 1.06; 95% CI, 0.99–1.14) or Model 3 (PR, 1.04; 95% CI, 0.97–1.11; Supplementary Table S9A).

Among eligible adult MEC participants, marginal missingness was greatest for PIR in 2017–March 2020 (14.0%) and alcohol use in August 2021–August 2023 (14.1%; Supplementary Table S1). In the covariate multiple-imputation analysis involving 11,516 participants, the association with kidney abnormality remained similar to the complete-case results (Model 2: OR, 1.16; 95% CI, 1.08–1.25; Model 3: OR, 1.12; 95% CI, 1.04–1.21). Positive associations with albuminuria were also observed in Model 2 (OR, 1.21; 95% CI, 1.13–1.30) and Model 3 (OR, 1.17; 95% CI, 1.08–1.26). For reduced eGFR, the association reached nominal statistical significance in Model 2 (OR, 1.10; 95% CI, 1.01–1.20; *p* = 0.030), whereas the Model 3 estimate was not statistically significant (OR, 1.07; 95% CI, 0.98–1.16; *p* = 0.115; Supplementary Table S9B).

Response-probability-weighted analyses also produced comparable estimates (Model 2: OR, 1.17; 95% CI, 1.09–1.25; Model 3: OR, 1.13; 95% CI, 1.05–1.21). Albuminuria estimates remained positive in Model 2 (OR, 1.21; 95% CI, 1.14–1.29) and Model 3 (OR, 1.17; 95% CI, 1.09–1.25), while the corresponding reduced-eGFR estimates were not statistically significant (Model 2: OR, 1.09; 95% CI, 0.99–1.19; Model 3: OR, 1.05; 95% CI, 0.97–1.15; Supplementary Table S9C).

## 4. Discussion

In this survey-weighted cross-sectional study, higher LAA5 was associated with greater odds of laboratory-defined kidney abnormality and albuminuria when modeled continuously. These associations were modest and persisted after expanded cardiometabolic adjustment. Findings for reduced eGFR were less conclusive and varied across exposure specifications, adjustment models, and covariate imputation.

Previous studies suggest that liver–kidney associations vary across hepatic phenotypes and renal outcomes. Elastography-defined fibrosis has been associated with albuminuria and incident CKD [4,5]. Evidence for GGT also differs by renal endpoint, with reported associations with albuminuria or persistent dipstick proteinuria [6,17] and an inconclusive adjusted association with incident CKD in another cohort [8]. Findings for imaging-defined steatosis have been less consistent, including inconclusive associations after cardiometabolic adjustment in population-based studies [7,26] and during follow-up of older adults with type 2 diabetes [27]. These marker-specific findings leave open how an age-referenced combination of structural and biochemical hepatic measurements relates to kidney abnormalities. The present study addresses this question by examining LAA5 alongside its constituent markers within a common survey sample.

Albuminuria and reduced eGFR represent complementary kidney dimensions [12,13]. In a type 2 diabetes cohort, steatosis-related scores were associated with albuminuria category progression, whereas fibrosis-related scores were associated with an eGFR decline of at least 40% [18]. This supports separate assessment of renal outcomes but does not establish phenotype-specific LAA5 associations. Our multinomial models identified positive associations with albuminuria only and both abnormalities, but overall coefficient heterogeneity was inconclusive, and the nominal albuminuria-only versus reduced-eGFR-only contrast was sensitive to adjustment (Supplementary Table S7). The reported albuminuria-only versus reduced-eGFR-only comparisons were nominal and are not interpreted as establishing a stronger LAA5 association with albuminuria only after accounting for multiple comparisons. Differences in statistical significance between individual phenotype associations do not themselves establish a between-phenotype difference, and inconclusive comparisons do not demonstrate equivalence. These comparisons concern mutually exclusive phenotypes rather than overlapping binary outcomes. Nominal reduced-eGFR findings in the Model 2 quartile and covariate-imputation analyses also caution against interpreting the inconclusive continuous estimates as absence of association (Table 2 and Supplementary Table S9B).

The composite requires particular interpretive care. LAI-5 translates sex-specific mortality-model estimates into age-equivalent years; LAA5 is the resulting age estimate minus chronological age [10]. Negative coefficients for CAP, ALT, and AST and positive coefficients for LSM and GGT mean identical LAA5 values can reflect different biomarker profiles; the original age terms also leave residual age dependence (Supplementary Table S2). In our separately fitted component models, LSM and GGT were associated with albuminuria after expanded adjustment, whereas the GGT–composite association was less conclusive in Model 3. A small Japanese cohort with type 2 diabetes and NAFLD linked LSM to one-year changes in log-transformed urinary albumin-to-creatinine measurements [28], whereas a separate small cross-sectional sample with type 2 diabetes found an inconclusive LSM–UACR correlation [18]. Our component models do not partition the composite association into independent contributions or establish incremental predictive value. The isolated inverse ALT–reduced-eGFR association remains exploratory (Figure 3 and Supplementary Table S4).

Shared cardiometabolic determinants offer a plausible context, but this study did not measure a specific metabolic or vascular pathway. BMI, diabetes, hypertension, and CVD may capture shared determinants; under alternative temporal assumptions, some may also lie downstream, making overadjustment possible [21]. Model 2 represents the primary multivariable association, while Model 3 provides an expanded conditional association. Changes in ORs across adjustment sets may also reflect noncollapsibility [29] and cannot quantify mediation. Neither model identifies a total or direct causal effect. Reverse directionality and residual confounding by incompletely characterized health conditions and treatments remain possible.

The exploratory analyses further delimit the findings. Three and four knot splines supported overall associations for the composite outcome and albuminuria, but not clearly for reduced eGFR; neither specification provided clear evidence of nonlinearity (Figure 2 and Supplementary Figure S3). Estimates at the exposure tails were imprecise, and these tests establish neither linearity nor a clinical threshold. The nominal sex interaction was not significant after FDR correction, and the other subgroup and disease-burden analyses did not establish effect modification. These findings do not prove homogeneous associations. Multiple testing across outcomes, components, exposure specifications, and subgroups increases the possibility of chance findings [23,30].

Strengths include evaluating the composite and its components within a common sample, formally comparing mutually exclusive phenotypes, and examining alternative modeling and missing-data assumptions. Nevertheless, measurement availability, elastography-quality requirements, and covariate completeness restricted the complete-case sample to 9276 of 14,608 adult MEC participants. Covariate imputation included only the 11,516 participants with observed exposure and outcomes. It did not recover missing liver or kidney measurements or address selection arising from missing or invalid exposure or outcome measurements or failure to meet elastography-quality requirements. Its validity depends on appropriate imputation models and missingness assumptions [31]. Response-probability-weighted associations were estimated among 9276 complete cases; their validity depends on a correctly specified inclusion model, a conditional missing-at-random assumption given the observed model predictors, and positive inclusion probabilities [25]. Selection related to unmeasured factors or departures from these assumptions may therefore remain. For the composite outcome and albuminuria, similar estimates across sensitivity analyses support stability only under the assumptions examined and do not demonstrate that selection bias has been eliminated. Original survey weights do not automatically correct analytical selection, and generalization beyond the analytical sample also depends on the selection mechanisms and appropriate pooling of the two survey releases.

Measurement and applicability also warrant caution. Single UACR and creatinine measurements cannot confirm persistence for at least three months [12,32]. UACR may be influenced by urinary creatinine related to muscle mass [33], while nonrenal determinants of serum creatinine can influence creatinine-based eGFR [34,35]. Their contribution to the present findings is unknown without direct muscle assessment or cystatin C-based evaluation; substituting waist circumference or another creatinine equation cannot resolve these concerns. Hyperfiltration was not assessed, and public-use age top-coding may affect LAA5 and eGFR calculations in the oldest participants. The original development and validation study involved adults aged ≥45 years and included NHANES data overlapping the present survey period [10]. Similar findings after flexible age adjustment or restriction to older adults do not establish transportability across age groups or measurement settings.

Finally, LAA5 is a model-derived hepatic composite expressed relative to chronological age [10], rather than a direct measurement of hepatic biological aging or its longitudinal rate. Predictive performance was not assessed in this study, and the observed cross-sectional associations do not establish that LAA5 can predict future kidney disease or provide incremental predictive value beyond the constituent biomarkers. The clinical meaning of LAA5 for kidney outcomes remains unestablished, and these findings do not demonstrate utility for kidney disease screening, risk stratification, or clinical decision-making. PR analyses supported the direction of the composite kidney outcome and albuminuria associations, but ORs and PRs are not interchangeable, and neither represents incident disease risk in this cross-sectional design [36]. Prospective studies with repeated kidney measurements are needed to evaluate longitudinal associations. Independent validation and formal comparisons with constituent biomarkers would additionally be required to assess predictive value for kidney outcomes.

## 5. Conclusions

In this cross-sectional NHANES analysis, higher LAA5 was associated with higher odds of kidney abnormality and albuminuria after multivariable adjustment. Evidence for an association with reduced eGFR was less consistent across analyses. These findings do not establish that LAA5 can predict future kidney disease. Independent prospective studies with repeated kidney measurements are needed to evaluate longitudinal associations and the persistence of kidney abnormalities.

## Figures and Tables

**Table 1:** Baseline characteristics of participants across LAA5 quartiles.

Characteristic	Overall	Q1	Q2	Q3	Q4	*p*-Value
Age, years	48.19 (16.91)	51.31 (16.64)	49.48 (16.72)	46.08 (16.90)	45.35 (16.69)	<0.001
LAA5, years	−1.22 (2.76)	−4.44 (1.34)	−1.90 (0.47)	−0.35 (0.46)	2.41 (2.02)	<0.001
CAP, dB/m	261.0 [217.0, 307.0]	297.0 [261.0, 338.0]	265.0 [229.0, 306.0]	241.0 [208.0, 282.0]	219.0 [184.0, 275.0]	<0.001
LSM, kPa	4.9 [4.1, 6.1]	4.6 [3.8, 5.7]	4.7 [3.9, 5.8]	5.0 [4.1, 6.0]	5.7 [4.5, 7.6]	<0.001
ALT, U/L	18.0 [14.0, 27.0]	22.0 [17.0, 31.0]	18.0 [14.0, 25.0]	16.0 [12.0, 22.0]	17.0 [12.0, 26.0]	<0.001
AST, U/L	20.0 [16.0, 24.0]	21.0 [18.0, 27.0]	19.0 [16.0, 23.0]	18.0 [16.0, 23.0]	19.0 [16.0, 24.0]	<0.001
GGT, U/L	20.0 [14.0, 31.0]	18.0 [13.0, 25.0]	18.0 [13.0, 26.0]	19.0 [13.0, 29.0]	31.0 [19.0, 56.0]	<0.001
eGFR, mL/min/1.73 m^2^	96.05 (20.26)	93.32 (19.68)	95.61 (20.08)	97.65 (20.09)	98.03 (20.95)	<0.001
UACR, mg/g	6.8 [4.6, 12.1]	6.3 [4.2, 11.2]	6.9 [4.7, 12.2]	6.8 [4.7, 11.7]	7.4 [4.7, 13.2]	<0.001
Sex						<0.001
Female	4845 (50.6)	490 (20.8)	1347 (55.6)	1613 (70.0)	1395 (58.9)	
Male	4431 (49.4)	1830 (79.2)	971 (44.4)	706 (30.0)	924 (41.1)	
Race/ethnicity						<0.001
Mexican American	886 (7.4)	244 (7.7)	249 (7.9)	209 (7.3)	184 (6.6)	
Non-Hispanic Black	1765 (10.0)	285 (5.7)	292 (6.1)	479 (11.1)	709 (18.0)	
Non-Hispanic White	4387 (65.0)	1260 (71.3)	1187 (67.3)	1053 (63.8)	887 (56.6)	
Other Hispanic	908 (7.6)	208 (6.3)	244 (8.3)	227 (7.3)	229 (8.4)	
Other race, including multiracial	1330 (10.0)	323 (8.9)	346 (10.4)	351 (10.5)	310 (10.4)	
Education						0.149
Less than high school	1280 (9.0)	317 (8.4)	311 (9.2)	294 (8.6)	358 (9.8)	
High school graduate/GED	2008 (25.2)	478 (24.5)	485 (24.7)	504 (23.9)	541 (28.0)	
Some college/AA degree	3043 (31.0)	724 (30.0)	762 (32.3)	764 (30.5)	793 (31.2)	
College graduate or above	2945 (34.8)	801 (37.1)	760 (33.8)	757 (37.0)	627 (30.9)	
PIR						<0.001
<1.3	2183 (17.3)	456 (14.1)	464 (14.8)	571 (18.2)	692 (22.8)	
1.3 to <3.5	3510 (35.0)	821 (31.9)	889 (34.8)	903 (36.5)	897 (37.3)	
3.5+	3583 (47.7)	1043 (54.0)	965 (50.4)	845 (45.3)	730 (40.0)	
BMI, kg/m^2^	29.55 (6.86)	30.44 (5.56)	29.41 (6.64)	29.03 (7.13)	29.21 (7.98)	<0.001
Smoking status						<0.001
Never	5413 (59.3)	1300 (57.1)	1395 (58.7)	1396 (62.3)	1322 (59.3)	
Former	2339 (25.3)	753 (32.3)	564 (26.1)	530 (21.1)	492 (20.7)	
Current	1524 (15.4)	267 (10.6)	359 (15.2)	393 (16.6)	505 (20.0)	
Alcohol use						0.107
Never	768 (7.0)	173 (6.5)	213 (7.3)	196 (6.9)	186 (7.1)	
Former	1631 (14.4)	458 (16.2)	423 (15.4)	369 (12.6)	381 (13.0)	
Current	6877 (78.7)	1689 (77.3)	1682 (77.3)	1754 (80.5)	1752 (79.9)	
Diabetes status						<0.001
No	6923 (78.7)	1630 (74.3)	1751 (79.3)	1822 (84.1)	1720 (77.6)	
PreDM	639 (6.8)	202 (9.1)	164 (7.1)	138 (5.0)	135 (5.7)	
Diabetes	1714 (14.4)	488 (16.6)	403 (13.6)	359 (10.9)	464 (16.7)	
Hypertension						0.004
No	4069 (50.0)	912 (46.1)	1035 (49.6)	1091 (54.1)	1031 (50.5)	
Yes	5207 (50.0)	1408 (53.9)	1283 (50.4)	1228 (45.9)	1288 (49.5)	
CVD						0.004
No	8299 (91.8)	2023 (89.6)	2105 (93.0)	2107 (93.1)	2064 (91.6)	
Yes	977 (8.2)	297 (10.4)	213 (7.0)	212 (6.9)	255 (8.4)	

Abbreviations: ALT, alanine aminotransferase; AST, aspartate aminotransferase; BMI, body mass index; CAP, controlled attenuation parameter; CVD, cardiovascular disease; eGFR, estimated glomerular filtration rate; GED, General Educational Development; GGT, gamma-glutamyl transferase; LAA5, LAI-5-derived liver age acceleration; LSM, liver stiffness measurement; PIR, poverty income ratio; PreDM, prediabetes; UACR, urinary albumin-to-creatinine ratio; LAA5 is a model-derived age difference (LAI-5 minus chronological age, in years) calculated using CAP, LSM, ALT, AST, and GGT, with chronological age entering both model predictors and sex determining the applicable parameters. The remaining characteristics presented in this table are not inputs to the LAA5 calculation. Differences in the component biomarkers across LAA5 quartiles partly reflect the construction of the index. LAA5 quartiles: Q1 and Q4 denote the lowest and highest LAA5 quartiles, respectively, based on the pooled unweighted study distribution. These categories indicate relative LAA5 levels, and their boundaries do not represent validated biological or clinical thresholds. Data are presented as mean (SD), median (IQR), or unweighted *n* (survey-weighted %), as applicable.

**Table 2:** Associations of LAA5 with kidney abnormality, albuminuria, and reduced eGFR.

	Model 1		Model 2		Model 3	
Characteristics	OR (95% CI)	*p*-Value	OR (95% CI)	*p*-Value	OR (95% CI)	*p*-Value
Kidney abnormality						
N/cases	9276/1521		9276/1521		9276/1521	
Per 1-SD increase	1.16 (1.08, 1.25)	<0.001	1.16 (1.08, 1.25)	<0.001	1.12 (1.04, 1.21)	0.003
Per 1-year increase	1.05 (1.03, 1.08)	<0.001	1.05 (1.03, 1.08)	<0.001	1.04 (1.02, 1.07)	0.003
Q1	Reference		Reference		Reference	
Q2	1.02 (0.75, 1.38)	0.921	1.04 (0.75, 1.44)	0.815	1.02 (0.74, 1.42)	0.892
Q3	1.09 (0.83, 1.45)	0.523	1.14 (0.85, 1.53)	0.383	1.10 (0.82, 1.48)	0.522
Q4	1.48 (1.15, 1.91)	0.003	1.47 (1.13, 1.91)	0.005	1.32 (1.01, 1.73)	0.040
*p* for trend		0.003		0.003		0.026
Albuminuria						
N/cases	9276/1081		9276/1081		9276/1081	
Per 1-SD increase	1.22 (1.14, 1.31)	<0.001	1.21 (1.13, 1.30)	<0.001	1.17 (1.09, 1.26)	<0.001
Per 1-year increase	1.07 (1.05, 1.10)	<0.001	1.07 (1.04, 1.10)	<0.001	1.06 (1.03, 1.08)	<0.001
Q1	Reference		Reference		Reference	
Q2	1.00 (0.71, 1.43)	0.979	1.02 (0.71, 1.47)	0.894	1.01 (0.70, 1.45)	0.966
Q3	1.08 (0.76, 1.53)	0.659	1.10 (0.77, 1.58)	0.577	1.07 (0.74, 1.55)	0.701
Q4	1.54 (1.16, 2.05)	0.004	1.52 (1.13, 2.03)	0.007	1.34 (0.99, 1.83)	0.059
*p* for trend		0.002		0.003		0.033
Reduced eGFR						
N/cases	9276/668		9276/668		9276/668	
Per 1-SD increase	1.06 (0.97, 1.17)	0.202	1.08 (0.98, 1.19)	0.103	1.05 (0.96, 1.15)	0.294
Per 1-year increase	1.02 (0.99, 1.06)	0.202	1.03 (0.99, 1.06)	0.103	1.02 (0.99, 1.05)	0.294
Q1	Reference		Reference		Reference	
Q2	1.00 (0.72, 1.39)	0.990	1.05 (0.73, 1.51)	0.791	1.04 (0.73, 1.46)	0.833
Q3	1.11 (0.82, 1.52)	0.485	1.22 (0.88, 1.69)	0.223	1.17 (0.86, 1.60)	0.312
Q4	1.31 (1.00, 1.72)	0.054	1.37 (1.03, 1.81)	0.032	1.26 (0.97, 1.64)	0.085
*p* for trend		0.059		0.026		0.072

Model 1: age, sex, and race/ethnicity. Model 2: Model 1 plus education, categorical PIR, BMI, smoking, and categorical alcohol use. Model 3: Model 2 plus diabetes status, hypertension, and CVD. Kidney abnormality denotes a single-visit kidney abnormality (UACR ≥30 mg/g or eGFR <60 mL/min/1.73 m^2^); persistence for ≥3 months cannot be established. Albuminuria: UACR ≥30 mg/g. Reduced eGFR: eGFR <60 mL/min/1.73 m^2^.

## Data Availability

The source data for this study are publicly available from the National Center for Health Statistics (NCHS) through two National Health and Nutrition Examination Survey (NHANES) releases: 2017–March 2020 Pre-Pandemic, https://wwwn.cdc.gov/nchs/nhanes/continuousnhanes/default.aspx?Cycle=2017-2020 (accessed on 24 May 2026), and August 2021–August 2023, https://wwwn.cdc.gov/nchs/nhanes/continuousnhanes/default.aspx?Cycle=2021-2023 (accessed on 24 May 2026). The analytic dataset was constructed from these public-use files as described in the Methods and Supplementary Methods.

## Supplementary Materials

The following supporting information can be downloaded at: https://www.mdpi.com/article/10.3390/healthcare14213759/s1, Figure S1A: Alternative directed acyclic graphs illustrating assumptions underlying covariate adjustment for the association between liver age acceleration (LAA5) and kidney abnormalities; Figure S1B: Alternative directed acyclic graphs illustrating assumptions underlying covariate adjustment for the association between liver age acceleration (LAA5) and kidney abnormalities; Figure S2: Age Dependence and Distribution of LAA5; Figure S3: Sensitivity of Restricted Cubic Spline Associations to the Number of Knots; Figure S4: Overlapping Cardiometabolic Comorbidities; Figure S5: Sensitivity Analyses of LAA5 and Kidney Abnormality; Table S1: Participant Selection and Completeness of Analysis Variables; Table S2: Source Parameters for the LAA5 Calculation; Table S3: Distribution of LAA5 in the Overall Cohort and Kidney Phenotypes; Table S4: LAA5 and Individual Liver Component Associations; Table S5: Exploratory Subgroup Associations with Kidney Abnormality; Table S6: Cardiometabolic Comorbidity Burden and Kidney Abnormalities; Table S7: Association of Liver Age Acceleration with Mutually Exclusive Kidney Abnormality Phenotypes; Table S8: Sensitivity Analyses of LAA5 and Kidney Abnormalities; Table S9: Alternative Effect Measures and Missing Data Analyses. Ref. [10] is cited in the supplementary.

## Abbreviations

The following abbreviations are used in this manuscript:

AA	Associate of Arts
ALT	alanine aminotransferase
AST	aspartate aminotransferase
BMI	body mass index
CAP	controlled attenuation parameter
CI	confidence interval
CKD	chronic kidney disease
CKD-EPI	Chronic Kidney Disease Epidemiology Collaboration
CVD	cardiovascular disease
eGFR	estimated glomerular filtration rate
FAP	fat attenuation parameter
FDR	false discovery rate
GED	General Educational Development
GGT	gamma-glutamyl transferase
HDL	high-density lipoprotein
IQR	interquartile range
LAA5	LAI-5-derived liver age acceleration
LAI-5	Liver Aging Index based on five liver-related markers
LSM	liver stiffness measurement
MAFLD	metabolic dysfunction-associated fatty liver disease
MEC	mobile examination center
MRDT	mortality rate doubling time
NAFLD	nonalcoholic fatty liver disease
NCHS	National Center for Health Statistics
NHANES	National Health and Nutrition Examination Survey
OR	odds ratio
PIR	poverty income ratio
PR	prevalence ratio
PreDM	prediabetes
ROR	relative odds ratio
SD	standard deviation
UACR	urinary albumin-to-creatinine ratio
