# Supplementary material for: Liver Age Acceleration and Kidney Abnormalities Among Adult NHANES Participants: A Cross-Sectional Study
Source: Healthcare (Basel). 2026 Nov 4;14(21):3759. doi: 10.3390/healthcare14213759 (PMC13763071; doi:10.3390/healthcare14213759)
Supplement: Supplementary file 1 [file healthcare-14-03759-s001.zip › Supplementary File.pdf]

Supplementary Table S1. Participant Selection and Completeness of Analysis Variables

A. Sequential exclusions by survey period

2017–March 2020

Step	Before	Excluded	Remaining
MEC base	14,300	0	14,300
Age at least 20	14,300	5,756	8,544
FibroScan examination status	8,544	555	7,989
Fasting at least 3 hours	7,989	248	7,741
At least 10 valid measurements	7,741	180	7,561
IQR median quality	7,561	165	7,396
Observed CAP_raw	7,396	1	7,395
Observed LSM_raw	7,395	0	7,395
Observed ALT	7,395	486	6,909
Observed AST	6,909	28	6,881
Observed GGT	6,881	0	6,881
Observed serum_creatinine_mg_dl	6,881	0	6,881
Observed uACR_mg_g	6,881	66	6,815
Observed sex	6,815	0	6,815
Observed Race	6,815	0	6,815
Observed Education	6,815	8	6,807

Step	Before	Excluded	Remaining
Observed PIR	6,807	891	5,916
Observed BMI	5,916	39	5,877
Observed Smoking status	5,877	2	5,875
Observed Alcohol use	5,875	236	5,639

Supplementary Table S1. Continued

August 2021–August 2023

Step	Before	Excluded	Remaining
MEC base	8,860	0	8,860
Age at least 20	8,860	2,796	6,064
FibroScan examination status	6,064	360	5,704
Fasting at least 3 hours	5,704	106	5,598
At least 10 valid measurements	5,598	157	5,441
IQR median quality	5,441	164	5,277
Observed CAP_raw	5,277	1	5,276
Observed LSM_raw	5,276	0	5,276
Observed ALT	5,276	504	4,772
Observed AST	4,772	11	4,761
Observed GGT	4,761	0	4,761

Step	Before	Excluded	Remaining
Observed serum_creatinine_mg_dl	4,761	2	4,759
Observed uACR_mg_g	4,759	58	4,701
Observed sex	4,701	0	4,701
Observed Race	4,701	0	4,701
Observed Education	4,701	2	4,699
Observed PIR	4,699	596	4,103
Observed BMI	4,103	18	4,085
Observed Smoking status	4,085	5	4,080
Observed Alcohol use	4,080	442	3,638

Sequential counts do not overlap; zero-count exclusions are retained. The eligible adult MEC base is 14608; 9276 are included and 5332 excluded. After the listed steps, 1 participant with missing CVD status was additionally excluded from 2017–March 2020 (5639 − 1 = 5638); the 2021–2023 count remained 3638.

Supplementary Table S1. Continued

B. Marginal missingness among eligible adults

Survey period	Variable	Adult MEC No.	Missing No.	Missing %
2017–March 2020	Age	8544	0	0.0
2017–March 2020	CAP	8544	622	7.3
2017–March 2020	LSM	8544	621	7.3
2017–March 2020	ALT	8544	664	7.8
2017–March 2020	AST	8544	695	8.1

Survey period	Variable	Adult MEC No.	Missing No.	Missing %
2017–March 2020	GGT	8544	664	7.8
2017–March 2020	serum_creatinine_mg	8544	662	7.7
2017–March 2020	uACR_mg_g	8544	225	2.6
2017–March 2020	Sex	8544	0	0.0
2017–March 2020	Race	8544	0	0.0
2017–March 2020	Education	8544	13	0.2
2017–March 2020	PIR	8544	1195	14.0
2017–March 2020	BMI	8544	163	1.9
2017–March 2020	Smoking status	8544	4	0.0
2017–March 2020	Alcohol use	8544	576	6.7
2017–March 2020	DM	8544	88	1.0
2017–March 2020	Hypertension	8544	1	0.0
2017–March 2020	CVD	8544	1	0.0
August 2021–August 2023	Age	6064	0	0.0
August 2021–August 2023	CAP	6064	450	7.4
August 2021–August 2023	LSM	6064	449	7.4
August 2021–August 2023	ALT	6064	626	10.3
August 2021–August 2023	AST	6064	638	10.5

Survey period	Variable	Adult MEC No.	Missing No.	Missing %
August 2021–August 2023	GGT	6064	620	10.2
August 2021–August 2023	serum_creatinine_mg	6064	621	10.2
August 2021–August 2023	uACR_mg_g	6064	154	2.5
August 2021–August 2023	Sex	6064	0	0.0
August 2021–August 2023	Race	6064	0	0.0
August 2021–August 2023	Education	6064	4	0.1
August 2021–August 2023	PIR	6064	792	13.1
August 2021–August 2023	BMI	6064	94	1.6
August 2021–August 2023	Smoking status	6064	7	0.1
August 2021–August 2023	Alcohol use	6064	855	14.1
August 2021–August 2023	DM	6064	42	0.7
August 2021–August 2023	Hypertension	6064	0	0.0
August 2021–August 2023	CVD	6064	0	0.0

Marginal missingness overlaps between variables and must not be added to calculate exclusion totals.

Supplementary Table S2. Source Parameters for the LAA5 Calculation

Biomarker	Unit	Log mean	Log SD	Male coefficient	Female coefficient
CAP	dB/m	5.5071221923	0.1420367125	-0.1788114109	-0.0815917511
LSM	kPa	1.9427459692	0.3340647884	0.1447408612	0.1108570214
ALT	U/L	3.0325008209	0.5479442922	-0.0629680903	-0.1071219575
AST	U/L	3.1381081033	0.5452344978	-0.0937019558	-0.0495435376
GGT	U/L	3.1816713962	0.7738010956	0.3257307992	0.2708418271

Sex-specific biomarker centering constants

Biomarker	Male z center	Female z center
CAP	-0.0891832594	0.0489636086
LSM	0.054902003	-0.0301424304
ALT	0.1028497724	-0.0564668306
AST	0.0315900256	-0.0173436322
GGT	0.2334980761	-0.128195678

Age coefficients and mortality rate doubling time

Sex	Age center	Liver-model age coefficient	Age-only coefficient	MRDT
Male	65.9168209074	0.115066198	0.1169298	6.023899
Female	64.9203855128	0.1236448904	0.1270141	5.605951

Note. Natural logarithms are used. Full sex-specific equations and calculation parameters are provided in the Supplementary Methods.

Supplementary Table S3. Distribution of LAA5 in the Overall Cohort and Kidney Phenotypes

Population	No.	Weighted %	Weighted mean (SD), y	Weighted median (IQR), y	Observed range, y
Overall	9276	—	-1.22 (2.76)	-1.27 (-2.92–0.34)	-12.27 to 21.28
2017–March 2020	5638	—	-1.24 (2.72)	-1.27 (-2.93–0.34)	-10.15 to 21.28
2021–2023	3638	—	-1.17 (2.82)	-1.29 (-2.89–0.36)	-12.27 to 15.02
Neither abnormality	7755	86.9	-1.25 (2.71)	-1.29 (-2.92–0.31)	-10.15 to 14.63
Albuminuria only	853	7.8	-0.80 (3.02)	-0.97 (-2.74–0.86)	-8.16 to 21.28
Reduced eGFR only	440	3.7	-1.49 (2.92)	-1.54 (-3.12–0.03)	-12.27 to 13.60
Both abnormalities	228	1.6	-0.81 (3.54)	-1.08 (-3.01–0.85)	-8.09 to 14.81

Note. Weighted percentiles describe the distribution and are not the unweighted quartile boundaries used in regression. Regression boundaries are −2.764865575, −1.116115646, and 0.515175282 years. Estimates are per 1-SD higher LAA5 (2.8515 years).

Supplementary Table S4. LAA5 and Individual Liver Component Associations

Indicator	Model	Kidney abnormality OR (95% CI)	Albuminuria OR (95% CI)	Reduced eGFR OR (95% CI)
LAA5	Model 2	1.16 (1.08–1.25)	1.21 (1.13–1.30)	1.08 (0.98–1.19)
	Model 3	1.12 (1.04–1.21)	1.17 (1.09–1.26)	1.05 (0.96–1.15)
CAP	Model 2	1.01 (0.89–1.16)	1.06 (0.91–1.22)	0.91 (0.80–1.04)
	Model 3	0.91 (0.81–1.03)	0.92 (0.81–1.04)	0.88 (0.78–1.01)
LSM	Model 2	1.19 (1.10–1.29)	1.26 (1.16–1.37)	1.00 (0.90–1.10)
	Model 3	1.12 (1.04–1.21)	1.18 (1.09–1.28)	0.97 (0.87–1.07)
ALT	Model 2	1.08 (0.95–1.23)	1.14 (0.99–1.32)	0.86 (0.75–0.99)
	Model 3	1.05 (0.93–1.20)	1.10 (0.96–1.26)	0.86 (0.75–0.99)
AST	Model 2	1.10 (0.98–1.23)	1.10 (0.97–1.25)	0.94 (0.82–1.09)
	Model 3	1.11 (1.00–1.24)	1.11 (0.99–1.25)	0.95 (0.82–1.10)
GGT	Model 2	1.17 (1.06–1.30)	1.24 (1.10–1.39)	1.02 (0.92–1.13)
	Model 3	1.10 (1.00–1.21)	1.14 (1.02–1.28)	0.98 (0.89–1.08)

Note. Components are natural-log transformed and standardized with their own fixed main-cohort SD. Each indicator is fitted separately on identical model-specific samples. These comparisons do not test predictive superiority or incremental value. Model 1 adjusts for age, sex, and race/ethnicity. Model 2 additionally adjusts for education, poverty income ratio (<1.3, 1.3 to <3.5, or ≥3.5), BMI, smoking (never, former, or current), and alcohol use (never, former, or current). Model 3 additionally adjusts for diabetes, hypertension, and CVD.

Supplementary Table S5. Exploratory Subgroup Associations with Kidney Abnormality

Variable	Group	No.	Cases	OR (95% CI)	Interaction *p*	FDR *p*
Age	<60	5,691	523	1.18 (1.03–1.35)	0.807	0.917
	60+	3,585	998	1.15 (1.04–1.28)		
Sex	Female	4,845	790	1.33 (1.17–1.52)	0.027	0.192
	Male	4,431	731	1.09 (0.99–1.21)		
BMI	<25	2,419	315	1.13 (0.95–1.35)	0.916	0.917
	25 to <30	3,034	462	1.17 (1.01–1.36)		
	30+	3,823	744	1.19 (1.04–1.35)		
Diabetes status	no	6,923	801	1.14 (1.03–1.27)	0.917	0.917
	PreDM	639	101	1.22 (0.86–1.72)		
	DM	1,714	619	1.13 (0.98–1.30)		
Hypertension	no	4,069	315	1.17 (0.98–1.39)	0.828	0.917
	yes	5,207	1,206	1.14 (1.04–1.26)		
Survey period	2017–March 2020	5,638	991	1.19 (1.09–1.30)	0.440	0.917
	2021–2023	3,638	530	1.13 (1.01–1.26)		
CVD	no	8,299	1,140	1.16 (1.07–1.26)	0.583	0.917
	yes	977	381	1.12 (0.99–1.27)		

Note. Each factor is assessed with continuous LAA5 per fixed SD. Within-group ORs are contrasts from full survey-weighted interaction models with Model 2 covariates; the subgroup factor replaces its duplicate adjustment term when applicable. Multi-level interaction terms are tested jointly. BH-FDR adjusts seven factor-level tests. Differences in within-group significance do not establish interaction.

Supplementary Table S6. Cardiometabolic Comorbidity Burden and Kidney Abnormalities

Outcome	Burden	No.	Cases	Weighted %	OR (95% CI)	Interaction *p*
Kidney abnormality	0	3,410	200	45.0	1.11 (0.91–1.36)	0.888
	1	3,673	569	38.8	1.14 (1.00–1.30)	
	2-3	1,894	714	16.2	1.10 (0.98–1.24)	
Albuminuria	0	3,410	148	45.0	1.15 (0.91–1.43)	0.584
	1	3,673	380	38.8	1.24 (1.08–1.43)	
	2-3	1,894	526	16.2	1.14 (1.01–1.28)	
Reduced eGFR	0	3,410	59	45.0	0.96 (0.75–1.24)	0.842
	1	3,673	251	38.8	1.05 (0.85–1.29)	
	2-3	1,894	344	16.2	1.04 (0.90–1.19)	

Note. Of 9276 main-analysis participants, 8977 had classifiable burden and 299 had indeterminate burden. Groups 0, 1, and 2–3 contained 3410, 3673, and 1894 participants. Exact disease count was unknown for 317 people, and unknown status was not treated as absence. Models adjust for Model 2 covariates without separately adding the three disease variables. Slopes are joint-model contrasts; overall interaction tests have 2 numerator df. All three outcome tests are exploratory.

Supplementary Table S7. Association of Liver Age Acceleration with Mutually Exclusive Kidney Abnormality Phenotypes

Kidney phenotype or contrast	No.<br>Model 2 / Model 3	Model 2<br>ROR (95% CI)	Model 3<br>ROR (95% CI)
Neither abnormality	7755 / 7755	Reference	Reference
Albuminuria only	853 / 853	1.20 (1.10–1.31)	1.16 (1.05–1.27)
Reduced eGFR only	440 / 440	1.03 (0.89–1.19)	1.00 (0.87–1.16)
Both abnormalities	228 / 228	1.28 (1.13–1.46)	1.22 (1.10–1.37)
Ratio of RORs: albuminuria only vs reduced eGFR only		1.17 (1.00–1.35)	1.15 (0.99–1.34)
Overall heterogeneity *p* value		0.066	0.102
Primary coefficient comparison *p* value		0.043	0.070

Note. Models 2 and 3 both include 9276 participants. ROR, relative odds ratio, relative to neither abnormality. Albuminuria only and reduced eGFR only exclude the other abnormality. Kidney abnormality denotes UACR ≥30 mg/g or 2021 CKD-EPI creatinine eGFR <60 mL/min/1.73 m^2. Albuminuria and reduced eGFR use these component thresholds. Single measurements do not establish chronicity. Estimates are per 1-SD higher LAA5 (2.8515 years). Model 1 adjusts for age, sex, and race/ethnicity. Model 2 additionally adjusts for education, poverty income ratio (<1.3, 1.3 to <3.5, or ≥3.5), BMI, smoking (never, former, or current), and alcohol use (never, former, or current). Model 3 additionally adjusts for diabetes, hypertension, and CVD. Intervals use t (40), and the overall contrast uses F (2,40). The principal ratio is exp (βalbuminuria-only − βreduced-eGFR-only), with joint covariance, and is not a risk or prevalence ratio.

Supplementary Table S8. Sensitivity Analyses of LAA5 and Kidney Abnormalities.

Analysis	Model	No.	Cases	OR (95% CI)	*p*-Value
Additional TC and HDL-C	Model 3	9,273	1,521	1.13 (1.05–1.22)	0.002
Additional lipid-lowering medication	Model 3	9,260	1,514	1.12 (1.04–1.20)	0.005
Exclude heavy alcohol use	Model 3	7,435	1,295	1.10 (1.00–1.20)	0.049
Waist circumference replaces BMI	Model 3	9,152	1,477	1.12 (1.03–1.20)	0.005
Unified MEC weights	Model 2	9,276	1,521	1.16 (1.09–1.25)	< 0.001
Unified MEC weights	Model 3	9,276	1,521	1.13 (1.05–1.21)	0.002
Linear age	Model 2	9,276	1,521	1.16 (1.08–1.25)	< 0.001
Flexible age df=3	Model 2	9,276	1,521	1.16 (1.09–1.25)	< 0.001
Age ≥ 45	Model 2	5,864	1,298	1.19 (1.09–1.29)	< 0.001
Linear age	Model 3	9,276	1,521	1.12 (1.04–1.21)	0.003
Flexible age df=3	Model 3	9,276	1,521	1.12 (1.04–1.21)	0.003
Age ≥ 45	Model 3	5,864	1,298	1.15 (1.05–1.26)	0.005
Exclude viral hepatitis yes; unknown retained	Model 2	9,034	1,450	1.14 (1.05–1.23)	0.002
Exclude viral hepatitis yes; unknown retained	Model 3	9,034	1,450	1.10 (1.01–1.19)	0.022
Viral hepatitis no only	Model 2	8,976	1,437	1.13 (1.05–1.23)	0.002
Viral hepatitis no only	Model 3	8,976	1,437	1.10 (1.01–1.19)	0.023
2009 CKD-EPI creatinine equation	Model 2	9,276	1,594	1.15 (1.07–1.24)	< 0.001

Analysis	Model	No.	Cases	OR (95% CI)	*p*-Value
2009 CKD-EPI creatinine equation	Model 3	9,276	1,594	1.11 (1.03–1.20)	0.010

Supplementary Table S9. Alternative Effect Measures and Missing Data Analyses

A. Prevalence ratios

Outcome	Model	No.	Cases	PR (95% CI)	*p*-Value
Kidney abnormality	Model 2	9,276	1,521	1.12 (1.06–1.18)	< 0.001
	Model 3	9,276	1,521	1.09 (1.03–1.14)	0.002
Albuminuria	Model 2	9,276	1,081	1.18 (1.11–1.24)	< 0.001
	Model 3	9,276	1,081	1.13 (1.07–1.20)	< 0.001
Reduced eGFR	Model 2	9,276	668	1.06 (0.99–1.14)	0.098
	Model 3	9,276	668	1.04 (0.97–1.11)	0.302

B. Covariate multiple imputation

Outcome	Model	No.	Cases	OR (95% CI)	*p*-Value
Kidney abnormality	Model 2	11,516	1,953	1.16 (1.08–1.25)	< 0.001
	Model 3	11,516	1,953	1.12 (1.04–1.21)	0.003
Albuminuria	Model 2	11,516	1,408	1.21 (1.13–1.30)	< 0.001
	Model 3	11,516	1,408	1.17 (1.08–1.26)	< 0.001
Reduced eGFR	Model 2	11,516	845	1.10 (1.01–1.20)	0.030
	Model 3	11,516	845	1.07 (0.98–1.16)	0.115

C. Response probability weighting

Outcome	Model	No.	Cases	OR (95% CI)	*p*-Value
Kidney abnormality	Model 2	9,276	1,521	1.17 (1.09–1.25)	< 0.001
	Model 3	9,276	1,521	1.13 (1.05–1.21)	0.002
Albuminuria	Model 2	9,276	1,081	1.21 (1.14–1.29)	< 0.001
	Model 3	9,276	1,081	1.17 (1.09–1.25)	< 0.001
Reduced eGFR	Model 2	9,276	668	1.09 (0.99–1.19)	0.077
	Model 3	9,276	668	1.05 (0.97–1.15)	0.237

Note. PRs use survey-weighted quasi-Poisson models with Taylor variance, not incidence risk ratios. MI uses 20 imputations × 20 iterations in 11516 adults with observed exposure and outcomes; only covariates are imputed, with Rubin pooling.

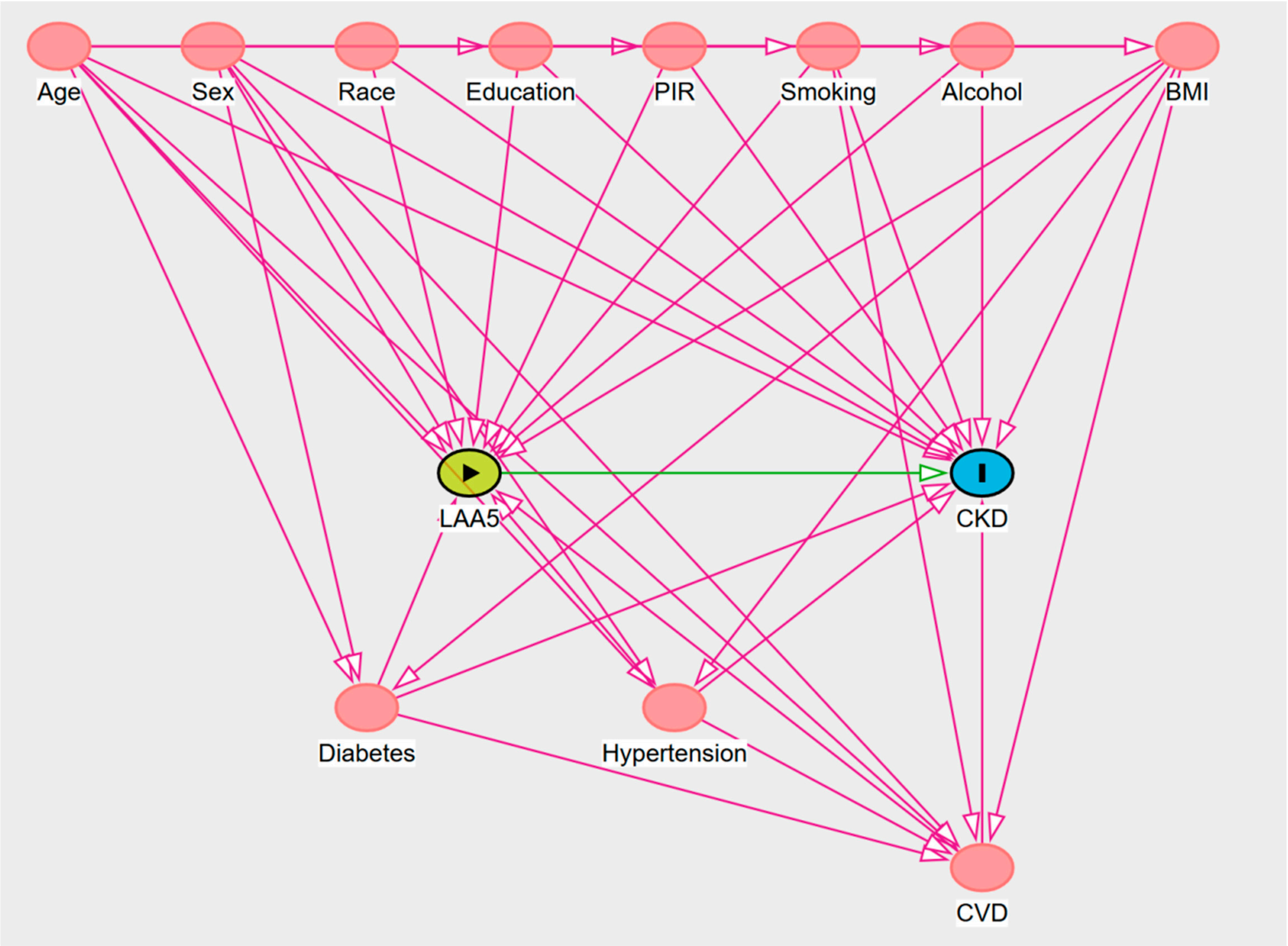

Supplementary Figure S1A： Alternative directed acyclic graphs illustrating assumptions underlying covariate adjustment for the association between liver age acceleration （LAA5） and kidney abnormalities.

Diabetes, hypertension, and cardiovascular disease (CVD) are represented as potential pre-existing common causes of liver-related measurements and kidney abnormalities.

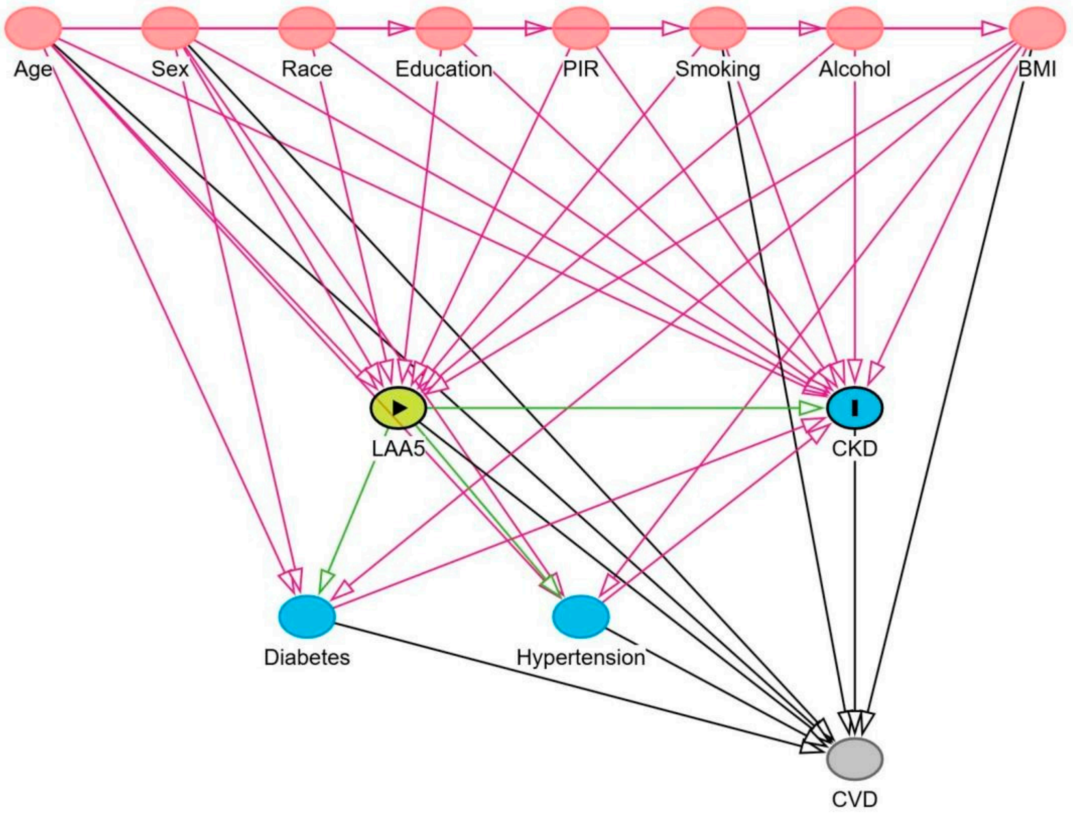

Supplementary Figure S1B： Alternative directed acyclic graphs illustrating assumptions underlying covariate adjustment for the association between liver age acceleration （LAA5） and kidney abnormalities.

An alternative causal structure considers these cardiometabolic conditions as potential downstream variables or components of related disease pathways. Both graphs retain the same demographic, socioeconomic, anthropometric, and lifestyle variables. The alternative assumptions inform the comparison between the primary multivariable model and the model additionally adjusted for diabetes, hypertension, and CVD. A separate sensitivity analysis restricted the study population to participants without these conditions.

Arrows represent hypothesized relationships rather than established causal effects. LAA5 is a derived measure of liver-related biological characteristics, and the cross-sectional study design does not permit identification of causal effects or confirmation of disease temporality.

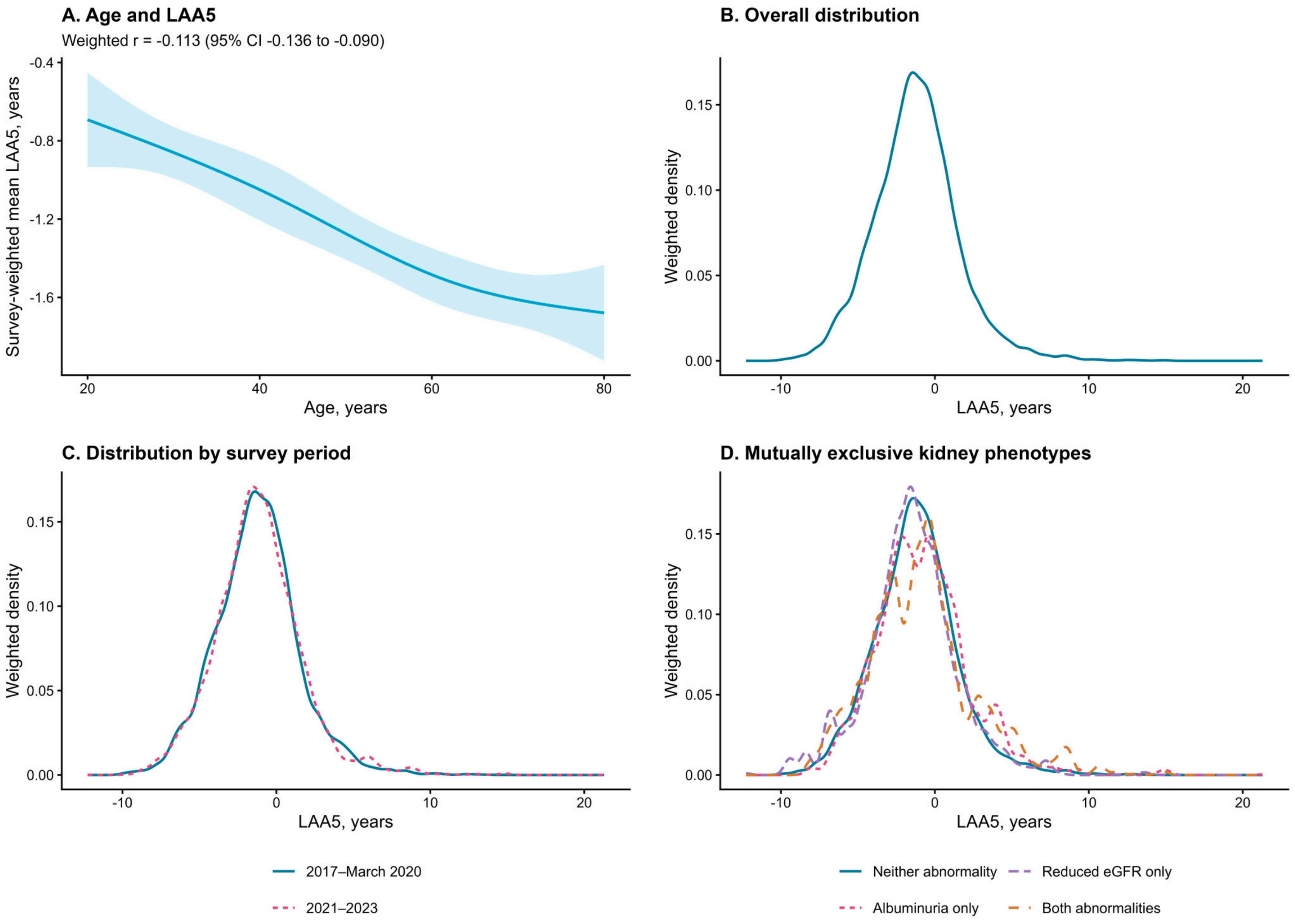

Supplementary Figure S2. Age Dependence and Distribution of LAA5

A, survey-weighted mean age trend with 95% CI; the correlation uses Taylor delta-method variance and a Fisher-z interval. B, overall survey-weighted density. All panels retain the same LAA5 formula and year scale. C and D, survey-weighted densities by survey period and mutually exclusive phenotype, with the same bandwidth as panel B. Kidney abnormality denotes UACR ≥30 mg/g or 2021 CKD-EPI creatinine eGFR <60 mL/min/1.73 m². Albuminuria and reduced eGFR use these component thresholds.

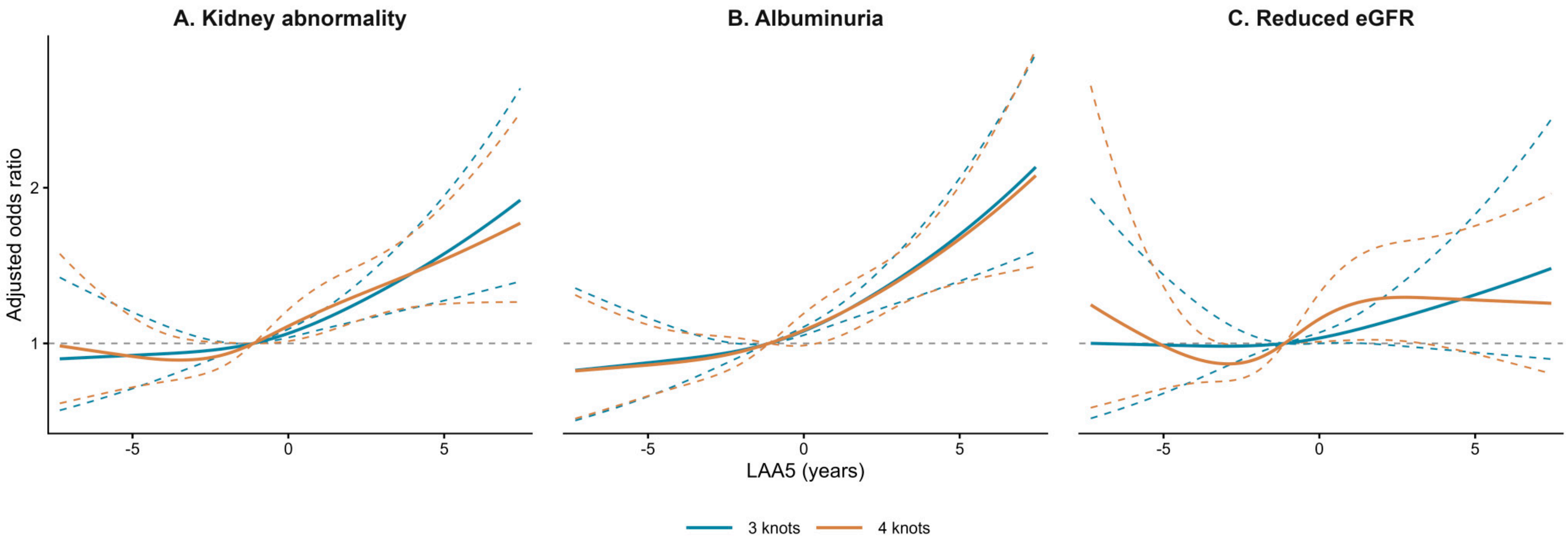

Supplementary Figure S3. Sensitivity of Restricted Cubic Spline Associations to the Number of Knots

Note. Solid lines show adjusted odds ratios; dashed colored lines show pointwise 95% confidence limits. Blue and orange denote 3 knots and 4 knots, respectively. Panels A–C represent kidney abnormality, albuminuria, and reduced eGFR. Both spline specifications use Model 2 covariates (age, sex, race/ethnicity, education, PIR categories, BMI, smoking, and alcohol use) and the same survey design, with 9276 participants and 40 design degrees of freedom. Design-adjusted Wald P values (overall / nonlinear): kidney abnormality 3 knots <0.001 / 0.235; kidney abnormality 4 knots <0.001 / 0.277; albuminuria 3 knots <0.001 / 0.268; albuminuria 4 knots <0.001 / 0.521; reduced eGFR 3 knots 0.113 / 0.543; reduced eGFR 4 knots 0.109 / 0.206.

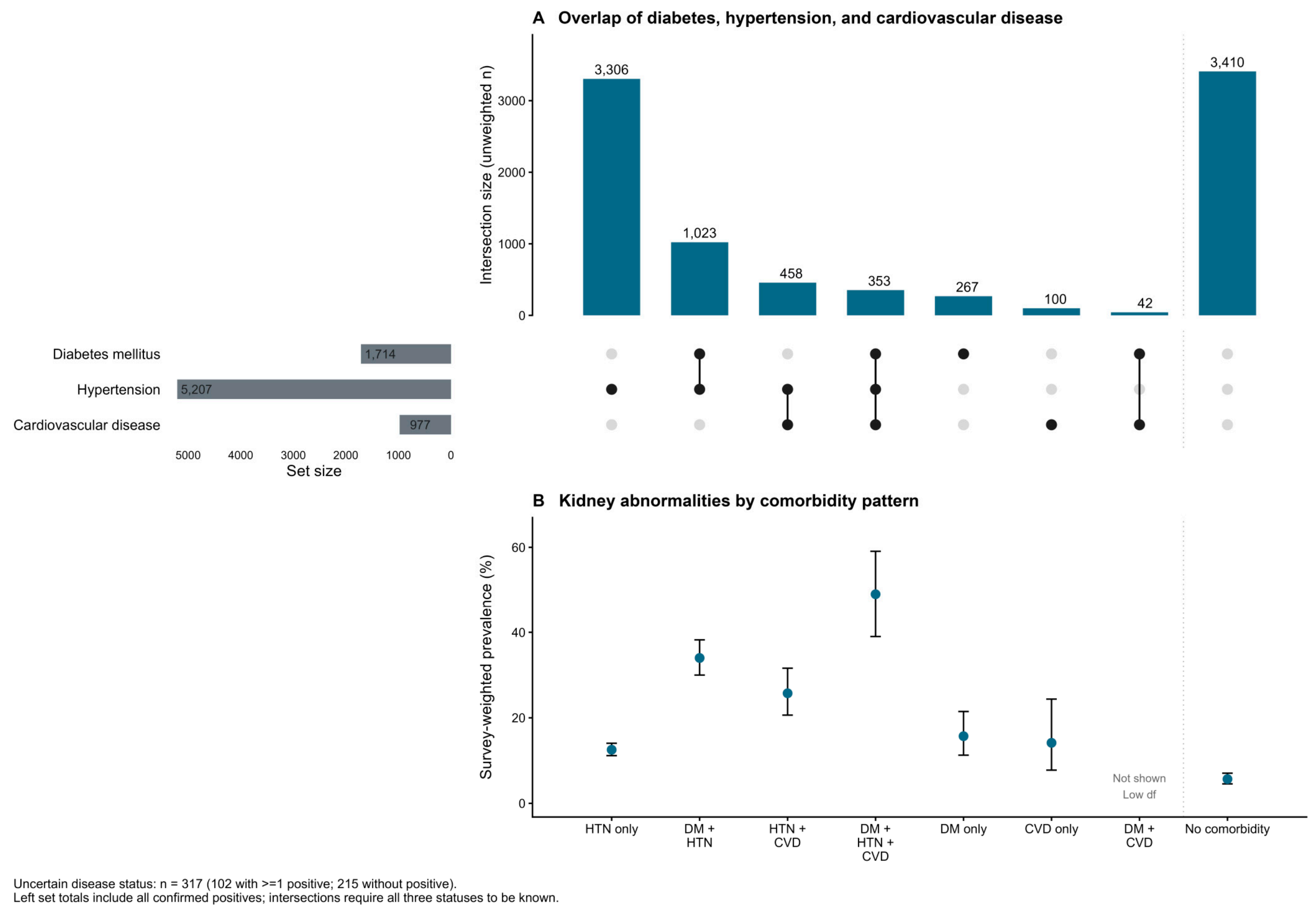

Supplementary Figure S4. Overlapping Cardiometabolic Comorbidities

A, unweighted intersections among 8959 participants with fully ascertained three-disease patterns; 317 uncertain patterns are counted separately. Diabetes excludes PreDM. Hypertension follows the implemented 130/80 mmHg definition; CVD includes CHF, CHD, angina, myocardial infarction, and stroke. B, survey-weighted kidney-abnormality prevalence with logit 95% CIs. DM+CVD-only prevalence is withheld because design df=5.

Sensitivity analysis	N	Cases	*p* value	Odds Ratio for Kidney Abnormality (95% CI)
Additional TC and HDL-C				
Model 3	9273	1521	0.002	1.13 (1.05–1.22)
Additional lipid-lowering medication				
Model 3	9260	1514	0.005	1.12 (1.04–1.20)
Exclude heavy alcohol use				
Model 3	7435	1295	0.049	1.10 (1.00–1.20)
Waist circumference replaces BMI				
Model 3	9152	1477	0.005	1.12 (1.03–1.20)
Unified MEC weights				
Model 2	9276	1521	<0.001	1.16 (1.09–1.25)
Model 3	9276	1521	0.002	1.13 (1.05–1.21)
Flexible age (natural spline, df=3)				
Model 2	9276	1521	<0.001	1.16 (1.09–1.25)
Model 3	9276	1521	0.003	1.12 (1.04–1.21)
Age ≥45 years				
Model 2	5864	1298	<0.001	1.19 (1.09–1.29)
Model 3	5864	1298	0.005	1.15 (1.05–1.26)
Viral hepatitis negative only				
Model 2	8976	1437	0.002	1.13 (1.05–1.23)
Model 3	8976	1437	0.023	1.10 (1.01–1.19)
2009 CKD-EPI creatinine equation				
Model 2	9276	1594	<0.001	1.15 (1.07–1.24)
Model 3	9276	1594	0.010	1.11 (1.03–1.20)

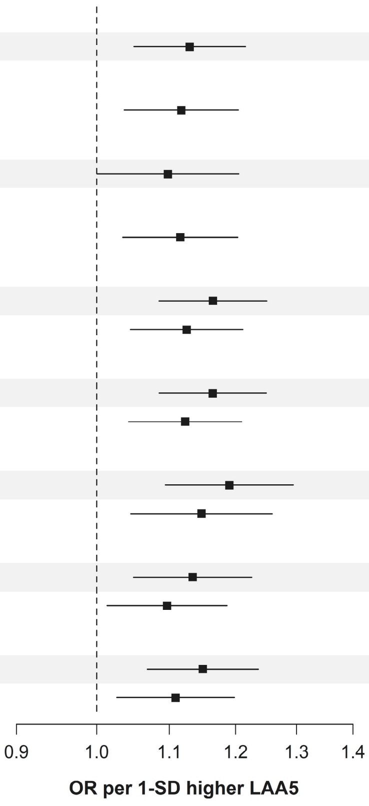

Supplementary Figure S5. Sensitivity Analyses of LAA5 and Kidney Abnormality

Model selection and rationale: Model 2 was the primary multivariable model, whereas Model 3 additionally adjusted for diabetes, hypertension, and cardiovascular disease. Analyses specifically designed to evaluate additional covariate adjustment or changes in covariate specification were reported under Model 3 as an expanded-adjustment sensitivity framework. For selected analyses examining survey-weight specification, age modelling, population restrictions, and alternative eGFR equations, both Models 2 and 3 were reported to evaluate the consistency of associations across adjustment levels. Other targeted sensitivity analyses were presented using Model 3.
