# Supplementary material for: Liver Age Acceleration and Kidney Abnormalities Among Adult NHANES Participants: A Cross-Sectional Study
Source: Healthcare (Basel). 2026 Nov 4;14(21):3759. doi: 10.3390/healthcare14213759 (PMC13763071; doi:10.3390/healthcare14213759)
Supplement: Supplementary file 1 [file healthcare-14-03759-s001.zip › Supplementary Methods.pdf]

Supplementary Methods: Calculation of LAI-5 and Liver Aging Acceleration

S1. Overview

The Liver Aging Index 5-variable model (LAI-5) is a model-derived index based on five liver-related structural and biochemical indicators: fat attenuation parameter (FAP), liver stiffness measurement (LSM), alanine aminotransferase (ALT), aspartate aminotransferase (AST), and gamma-glutamyl transferase (GGT). In NHANES transient elastography data, FAP was operationalized using median controlled attenuation parameter (CAP; dB/m). LSM was measured in kPa; ALT, AST, and GGT in U/L; and chronological age (CA) in years.

LAI-5 was implemented using the original authors' sex-specific Cox–Gompertz calculator, including sex-specific biomarker centering constants and full-precision coefficients. Liver aging acceleration (LAA5) was obtained from the difference between the liver-model and age-only-model predictors; LAI-5 equals CA plus LAA5 [1]. The index was applied without refitting or recalibration in NHANES.

S2. Variable transformation

For each participant, FAP/CAP, LSM, ALT, AST, and GGT were first transformed using the natural logarithm and then standardized as follows:

$$z_X = \frac{\ln(X) - \mu_X}{\sigma_X}$$

Here X denotes an input biomarker, μX its reference mean on the natural-log scale, and σX its reference standard deviation. This pooled standardization is followed by the sex-specific z-centering described in S4.

The transformation parameters used in this study are shown in table below.

Transformation parameters for LAI-5 biomarkers

Biomarker	NHANES operational variable	Unit	Log mean	Log SD
FAP	CAP	dB/m	5.5071221923	0.1420367125
LSM	LSM	kPa	1.9427459692	0.3340647884
ALT	ALT	U/L	3.0325008209	0.5479442922
AST	AST	U/L	3.1381081033	0.5452344978
GGT	GGT	U/L	3.1816713962	0.7738010956

Participants with missing, zero, or negative values for any LAI-5 input were assigned missing LAI-5 values because natural logarithmic transformation requires positive input values.

S3. Sex-specific age centering

Chronological age was centered by sex before being entered into the LAI-5 Cox-Gompertz linear predictor:

$$CA_{c,sex} = CA - \overline{CA}_{sex}$$

where $\overline{CA}_{sex}$ was 65.9168209074 years for men and 64.9203855128 years for women.

S4. Sex-specific LAI-5 liver-model linear predictors

In the displayed equations, biomarker z denotes sex-centered z* = z − c (not pooled z). In FAP, LSM, ALT, AST, GGT order, male c = (−0.0891832594, 0.0549020030, 0.1028497724, 0.0315900256, 0.2334980761); female c = (0.0489636086, −0.0301424304, −0.0564668306, −0.0173436322, −0.1281956780).

For men:

$$\eta_{LAI\text{-}5,male}=0.115066198\times CA_{c,male}-0.1788114109\times z_{FAP}+0.1447408612\times z_{LSM}$$
$$-0.0629680903\times z_{ALT}-0.0937019558\times z_{AST}+0.3257307992\times z_{GGT}$$

For women:

$$\eta_{LAI\text{-}5,female}=0.1236448904\times CA_{c,female}-0.0815917511\times z_{FAP}+0.1108570214\times z_{LSM}$$
$$-0.1071219575\times z_{ALT}-0.0495435376\times z_{AST}+0.2708418271\times z_{GGT}$$

S5. Sex-specific null-model linear predictors

The null model used centered chronological age only:

For men:

$$\eta_{0,male}=0.1169298\times CA_{c,male}$$

For women:

$$\eta_{0,female}=0.1270141\times CA_{c,female}$$

S6. Conversion of relative hazard to liver aging acceleration

The difference between the liver-model and null-model linear predictors was converted into a Gompertz-equivalent age difference, defined as liver aging acceleration from the LAI-5 model (LAA5):

$$LAA5_{sex}=\frac{\eta_{LAI\text{-}5,sex}-\eta_{0,sex}}{\ln(2)}\times MRDT_{sex}$$

Here MRDT is the sex-specific mortality rate doubling time: 6.023899 years for men and 5.605951 years for women (full author-calculator precision).

Accordingly, for men:

$$LAA5_{male}=\frac{\eta_{LAI\text{-}5,male}-\eta_{0,male}}{\ln(2)}\times 6.023899$$

For women:

$$LAA5_{female}=\frac{\eta_{LAI\text{-}5,female}-\eta_{0,female}}{\ln(2)}\times 5.605951$$

S7. Calculation of LAI-5

The final LAI-5 was calculated by adding LAA5 to chronological age:

$$LAI\text{-}5=CA+LAA5$$

Positive or negative LAA5 denotes model-derived age estimates above or below chronological age, not validated clinical thresholds.

S8. Exposure definitions used in statistical analyses

For regression, LAA5 was modeled continuously per fixed 1-SD increase and in pooled, unweighted sample quartiles. The corrected main cohort (N = 9,276) has unweighted SD = 2.8514855075001 years; this denominator was held fixed across models and sensitivity analyses.

$$LAA5_z = \frac{LAA5 - \overline{LAA5}}{SD(LAA5)}$$

Quartiles were defined using pooled unweighted boundaries of −2.764865575, −1.116115646, and 0.515175282 years. Q1–Q4 indicate sample-relative ranks rather than validated “decelerated” or “accelerated” liver aging groups. The ordinal Q1–Q4 code was used for exploratory trend testing.

S9. Parameter summary

Sex-specific LAI-5 Cox-Gompertz parameters

Sex	Parameter	Value
Men	Age (liver)	0.115066198
Men	CAP	−0.1788114109
Men	LSM	0.1447408612
Men	ALT	−0.0629680903
Men	AST	−0.0937019558
Men	GGT	0.3257307992
Men	Age (null)	0.1169298
Men	Age center	65.9168209074
Men	MRDT	6.023899
Women	Age (liver)	0.1236448904
Women	CAP	−0.0815917511
Women	LSM	0.1108570214
Women	ALT	−0.1071219575
Women	AST	−0.0495435376
Women	GGT	0.2708418271
Women	Age (null)	0.1270141
Women	Age center	64.9203855128
Women	MRDT	5.605951

S10. Quality control

Quality control checked sex, units, positive input values, log-standardization, sex-specific centers and full-precision coefficients. R calculations were checked against the author calculator. One fixed full-cohort SD was used across analyses.

S11. Reproducibility note

CAP operationalized FAP in NHANES. Author-calculator parameters were applied without recalibration. Because source development/validation largely involved adults ≥45 years, application at ages 20–44 has uncertain transportability; age ≥45 restriction was examined.

Reference

1. Wu, Z.; Wu, S.; Song, S.; Huang, Y.; Yu, C.; Sun, D.; Pei, P.; Yang, L.; Chen, Y.; Du, H.; et al. Liver Aging Index: A Noninvasive Score for Liver Biological Aging and Liver-Related Outcomes in Multicohorts. *Aging Cell* **2026**, *25*, e70565, doi:10.1111/acel.70565.
